# Assessing the impact of tungiasis on children’s quality of life in Kenya

**DOI:** 10.1371/journal.pntd.0012606

**Published:** 2025-09-08

**Authors:** Lynne Elson, Berrick Otieno, Abneel K. Matharu, Naomi Riithi, Esther Jebor Chongwo, Francis Mutebi, Hermann Feldmeier, Jürgen Krücken, Ulrike Fillinger, Amina Abubakar

**Affiliations:** 1 Kenya Medical Research Institute (KEMRI)-Wellcome Trust, Kilifi, Kenya; 2 Centre for Tropical Medicine and Global Health, Nuffield Department of Medicine, University of Oxford, Oxford, United Kingdom; 3 Institute for Human Development, Aga Khan University, Nairobi, Kenya; 4 Human Health Theme, International Centre of Insect Physiology and Ecology, Nairobi, Kenya; 5 Institute for Parasitology and Tropical Veterinary Medicine, Freie Universität Berlin, Berlin, Germany; 6 School of Veterinary Medicine and Animal Resources, College of Veterinary Medicine, Animal Resources and Biosecurity, Makerere University, Kampala, Uganda; 7 Institute of Microbiology, Infectious Diseases and Immunology, Charité University Medicine, Berlin, Germany; AstraZeneca plc, UNITED STATES OF AMERICA

## Abstract

Tungiasis is a neglected tropical skin disease caused by the sand flea, *Tunga penetrans* which penetrates the skin causing considerable pain and itching. In this cross-sectional study we aimed to assess its impact on the quality of life of school children in Kenya. School pupils (198) aged 8–14 years with tungiasis were randomly selected and interviewed using a tungiasis-specific quality of life instrument (TLQI). The caregivers of each infected pupil and 199 randomly selected caregivers of uninfected pupils were interviewed using the proxy KIDscreen52 to assess their child’s general health-related quality of life (HR-QoL). Generalized linear models were used to assess associations between quality-of-life variables, children’s tungiasis status and other covariables. Among infected children, 62.4% had TLQI scores reflecting a moderate to very high impact, with no significant difference between mild and severe cases. Severe cases had a lower proxy-HR-QoL than uninfected pupils (β -21.15, 95% CI -39.63 − -2.68, p = 0.025), but this was not significant in multivariable models. For the first time, this study demonstrated for children whose caregivers were depressed, tungiasis had a higher impact on their quality of life (TLQI adjusted β 0.28, 95% CI 0.08 − 0.49, p = 0.006) and had a lower general HR-QoL (adjusted β -40.34, 95%CI -55.91 − -24.76, p < 0.001). Conversely, if their caregiver showed them affection, tungiasis had a lower impact on their quality of life (TLQI, adjusted β -0.45, 95% CI -0.70 − -0.20, p < 0.001). Further studies are needed to investigate the interaction of tungiasis with parenting styles, the mental health of children and their caregivers and their effect on children’s well-being. However, this evidence indicates programs aiming to control tungiasis should include activities targeting the mental health and parenting style of caregivers.

## Author summary

Tungiasis is an extremely neglected tropical skin disease which causes immense suffering among children in resource poor communities in many tropical countries. A good quality of life is considered a right of all people and is consequently used as the most important outcome of treatment programs. The impact on quality of life by tungiasis has only been investigated in very few studies, but evidence of impact is needed to monitor interventions. Here we assessed the impact of the disease on the quality of life of school pupils using a disease-specific instrument with children and a general health-related instrument with their caregivers. The study demonstrated the considerable impact tungiasis has on the quality of life of school-age children. Tungiasis affected children’s ability to sleep and caused feelings of sadness and shame. Tungiasis had a greater impact on children whose caregivers were depressed, and they had a lower general health-related quality of life. Conversely, tungiasis had a lower impact on quality of life for children whose caregivers showed them affection. Further research is needed to investigate the interaction of tungiasis with parenting styles and the mental health of children and their caregivers and their effect on children’s well-being. Programs aiming to control tungiasis should include activities targeting the mental health and parenting style of caregivers.

## Background

Tungiasis is a neglected tropical skin disease which historically has received little or no attention for research, surveillance and interventions from national governments, their partners and donors. The lack of research has meant there is no highly effective treatment available in endemic areas and as a consequence patients do not seek health care at facilities and governments have been hamstrung to develop and deliver effective intervention programs. Tungiasis has now been added to the World Health Organization’s (WHO) list of Neglected Tropical Diseases (NTDs) targeted for control [1] and is included in the **“**Strategic Framework for Integrated Control and Management of Skin-Related Neglected Tropical Diseases” (SNTDs) [2] SNTDS are a sub-group of 10 NTDs with skin manifestations which impair, disfigure and disable the patient such as lymphatic filariasis, leprosy, cutaneous leishmaniasis, scabies and tungiasis. Apart from the physical symptoms, many patients experience stigma, discrimination, poor mental health and socioeconomic outcomes [3]. The SNTD framework encourages the integration of activities to address multiple diseases through a comprehensive approach including case detection, treatment, rehabilitation, stigma reduction and mental health services.

Tungiasis is caused by female sand fleas (*Tunga penetrans)* which penetrate the skin and stay embedded for their remaining life [4]. The disease mostly affects resource-poor communities across Latin America and sub-Saharan Africa [5]. The global burden of disease is unknown, but 668 million people (304 million in East Africa) have been estimated to be at risk of infection [6]. The only national survey ever conducted, was in Kenya, and estimated the national prevalence of tungiasis to be 1.3% [7] with the regions of the country most at risk being the coastal strip, the central highlands and the west [8]. The prevalence from small surveys within affected communities has been reported to range from 7% to 60% [9–12]. Children, particularly boys [7,12], elderly people and people with disabilities carry the highest disease burden [13].

The embedded flea causes severe morbidity with considerable pain and itching resulting from an intense inflammatory response around the rapidly growing flea [14] exacerbated by bacterial superinfection of the lesions [15]. The extent of morbidity is positively correlated with the infection intensity, and having more than 10 fleas has been classified as severe tungiasis [16]. While fleas have been found on any location of the body that has been in contact with the soil (hands, elbows, knees, buttocks) the vast majority of the embedded sand fleas are located on the feet [5].

Little is known about the further impact of tungiasis infection on a patient’s life. The pain and itching caused by inflammation have been reported to affect children’s ability to sleep [14], walk [14,17], attend school [18,19] and pay attention in class, resulting in poorer school exam performance [18,19]. Recently a study in Kenya demonstrated tungiasis was associated with poor neurocognitive functioning including literacy, language, cognitive flexibility, working memory, response inhibition, numeracy and fine motor skills [20]. Two previous studies in Kenya using a tungiasis specific Quality of Life Index (TLQI) both demonstrated tungiasis significantly impacts a child’s quality of life [19,21].

The World Health Organization (WHO) defines quality of life as “an individual’s perception of their position in the life and context of their culture and in relation to their goals, expectations, standards and concerns” [22]. Health-related quality of life reflects the impact of disease on quality of life. Due to the globally accepted right of all people to a good quality of life, clinical trials and intervention programs are now using this as a secondary outcome measure in the treatment of various diseases such as cancer [23], diabetes [24] and lymphatic filariasis [25]. To monitor any interventions, suitable instruments are needed to measure the quality of life of patients and the impact of diseases. Various instruments have been developed and validated to assess general health-related quality of life in different populations, such as the WHOQOL [22], WHODAS2.0 [26] and the KIDSCREEN [27] for children and adolescents, while others are designed to be specific for certain diseases, for example the DLQI for any skin disease [28] and the LFSQQ for lymphatic filariasis [29].

The objective of this study was to determine the impact of tungiasis on children’s quality of life and to compare the impact of mild and severe disease in a community with a high disease burden using both the disease-specific TLQI and a general health related quality of life instrument (KIDSCREEN).

## Methods

### Ethics statement

This study was performed in line with the principles of the Declaration of Helsinki*.* The study was approved by the KEMRI Scientific and Ethics Review Committee(approval number non-KEMRI 644), as well as the Ethikkommission of the Charité Berlin (reference number EA2/100/16). Permission to conduct the study was also obtained from the county and sub-county Health Management Teams and the Department of Education. Written informed consent was obtained from the school Head Teachers and the parent teachers’ association (PTA) chairperson. Caregivers of the pupils selected for interviews gave written informed consent for their own involvement as well as for their child, all ages enrolled. In addition, pupils aged 12–14 years themselves gave signed written informed assent.

All data were collected on PIN protected electronic tablets, stored on password protected databases on the servers of the International Centre of Insect Physiology and Ecology, Nairobi. Personal identifiers were removed from analyses data sets.

All pupils with tungiasis were treated by the community health workers or the local health facility using benzyl benzoate, which was the product chosen by the county health managers and provided by the study free of charge

### Study design

This was a cross-sectional study of children aged 8–14 years, the age group most affected by tungiasis [9], plus their caregivers. Caregivers were classified as the adult who provided most of the care for the child, cooking for them, watching out for their safety and at a younger age bathing them.

### Study setting

**Fig 1:**
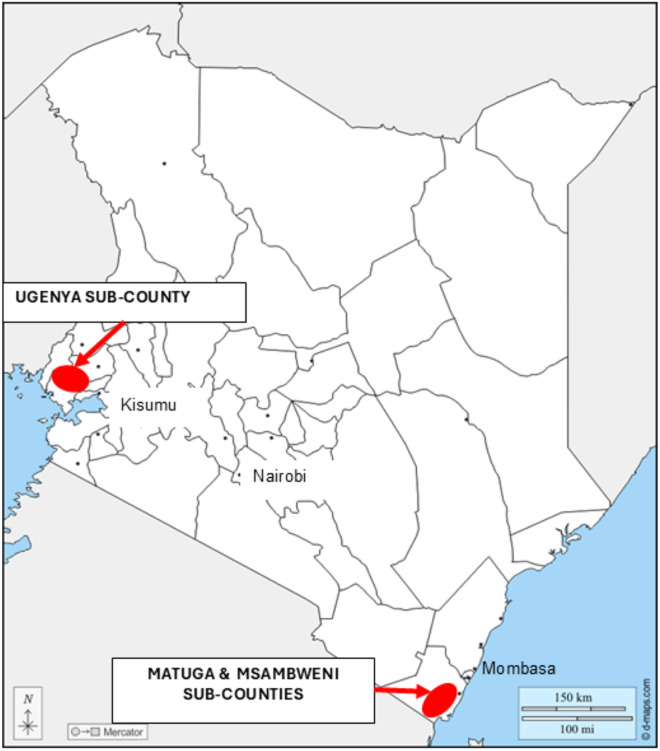
Map of Kenya showing the location of the sub-counties included in the study. Base map obtained from https://d-maps.com/carte.php?num_car=236&lang=en.

The study reported here is part of a larger study on the ecology and impact of tungiasis in two regions of Kenya having a high prevalence of tungiasis [16]. The recruitment and data collection were conducted between February 2020 and April 2021 in the sub-counties of Msambweni and Mutuga of Kwale County on the southeast coast, and in Ugenya sub-county of Siaya County in western Kenya (Fig 1).

### Sample size

The same group of pupils were enrolled for several impact assessments, both quality of life reported here, and neurocognitive function which was published previously [20]. The sample size was based on the mean difference in fluency scores for infected and uninfected pupils using a previous study in a similar setting with a mean category fluency of 15.97 [10]. Assuming a common standard deviation of 2, the study aimed to enroll 506 participants (253 infected and 253 uninfected) to detect at least 0.05 difference in means at *α *= 0.05 and power of 0.8. The sample size was calculated using Stata [11]. Inclusion criteria for infected pupils were having at least one embedded flea, living in a house with an unsealed earthen floor and the availability of a caregiver to provide informed consent and to also enroll for the assessments. In addition to those described for infected pupils, uninfected pupils had to have no signs of infection and no other infected family members to increase the certainty that they were not temporarily uninfected.

### Sampling strategy

To identify infected and uninfected pupils aged 8–14 years to enroll for the quality-of-life assessments, pupils in public primary schools were enrolled and examined for tungiasis. Public primary schools, 15 from Kwale and 20 from Siaya, were randomly selected from lists provided by the Departments of Education using the paper lottery method. In each school, 51 boys and 51 girls between the age of 8 and 14 years were quasi-randomly selected by lining the pupils up in three age groups; 8 and 9 years; 10 and 11 years; 12–14 years) and by sex within each age group. Every n^th^ (depending on the total number in the group) pupil was then selected in each sex and age group until 17 from each was reached, to a total of 102 in each school, 3,870 pupils from both counties. Each selected pupil was carefully examined for embedded fleas. For those found to be infected the number of embedded fleas (live, dead and manipulated lesions) were counted. After the examinations, using a paper-lottery method, in each school three boys and three girls were randomly selected from among tungiasis cases, and three boys and three girls from uninfected pupils, for a total of 210 cases and 210 uninfected pupils for the quality-of-life assessments. One caregiver for each of these children was also enrolled in the study.

### Procedures

The selected infected and uninfected pupils were first interviewed using structured questionnaires covering sociodemographic and psychosocial covariables about themselves, their family, their house, and school life. Only the infected pupils were then also interviewed to assess the impact of tungiasis on their quality of life using the Tungiasis Life Quality Index (TLQI). The caregivers of infected and uninfected pupils were interviewed for their perceptions of the health-related quality of life of their children using the proxy KIDSCREEN52^®^ (HR-QoL).

Through our previous work with families affected by tungiasis we had developed a hypothesis that caregiver mental health may be an underlying factor in child neglect and poor hygiene and therefore be a risk factor for tungiasis infection among children. Tungiasis has previously been associated with poor hygiene [12,30]. Consequently, the caregivers of enrolled pupils were all assessed for their own mental health using the Patient Health Questionnaire (PHQ-9) [31] and their stress level using the Parental Stress Scale (PSS) [32]. This also enabled us to investigate whether caregiver mental health may confound the impact of tungiasis on children’s quality of life.

### Tools for assessing the impact of tungiasis on QoL

#### The tungiasis-specific instrument (TLQI).

Life quality impairment was assessed using two different tools. One instrument was a tungiasis-specific tool inspired by the Children’s Dermatological Life Quality Index [33] which was adapted previously (TLQI) [21] and is the only quality of life instrument developed for patients with tungiasis. The instrument is provided in S1 Table. The original TLQI tool had six domains each with one question, as listed in Table 1 which asked: “During the last week rate the following according to the scales (not at all, only a little, quite a lot, very much): How embarrassed or ashamed did you feel because of the jiggers, How much did the jiggers make it difficult for you to walk/run, How much do the jiggers affect your concentration in class, affect your sleep, affect your friendships, and how much are other children mean/cruel/unkind to you”.

**Table 1:** Comparison of the instrument domains.

TLQI-self	KIDSCREEN52-Proxy
Mobility (walk/run)	Physical well-being
Feeling angry Feeling sad	Moods & emotions
Shame (embarrassed)	Self-perception
Friendships	Social support & peers
Concentration due to itching	School environment
Bullying	Social acceptance
Sleep disturbance	
	Psychological well-being
	Autonomy
	Parent relations
	Financial resources

Based on our personal experience of tungiasis and interacting with children with tungiasis we added two new questions; “how much do the fleas make you feel sad” and “how often do you feel angry” for a total of 8 domains. The maximum total score was 24. The higher the score, the higher the impact of tungiasis and the lower the quality of life. The instrument was piloted with a few pupils in the community who did not participate in the study. This instrument is not subject to licensing and has not been systematically validated.

#### Proxy-KIDSCREEN52.

The second instrument assessed the caregiver’s perception of the child’s general health-related quality of life (HR-QoL) through interviews with the caregiver using a standardized screening instrument for children and adolescents, the proxy-KIDSCREEN52 The instrument is provided in S2 Table [27]. This instrument was chosen for this study as it has been previously validated for use in South Africa [34], Uganda and Kenya [35,36], is available publicly for free in Kiswahili [37] which we needed, and specifically assesses the health-related quality of life of children in the age range of our target population. This tool has 52 questions across 10 domains covering physical wellbeing, psychological wellbeing, moods and emotions, self-perception, social support and peers, social acceptance, school environment, autonomy, parent relations and financial resources. Each domain in the KIDSCREEN52 instrument has between 3 and 7 questions with each being a 5-point Likert scale.

As illustrated in Table 1, this instrument has some conceptual overlaps with the tungiasis-specific instrument. For instance, the ability to walk and run is covered by physical well-being questions while some questions under moods and emotions address feeling sad and angry. Both tools included domains assessing a child’s experience of stigma and discrimination: feeling embarrassed or ashamed are similar to questions in self-perception, affect on friendships is similar to questions in social support, and bullying is included in the questions for social acceptance. Sleep disturbance is only covered in the TLQI while psychological well-being, autonomy, financial resources and parent relations are only covered in the KIDSCREEN52 instrument.

Both tools were translated from English into the local language, Kidigo or Kiswahili in Kwale, Dholuo or Kiswahili in Siaya.

The internal consistency of each instrument was tested using Cronbach’s alpha and found both instruments had good internal consistency in this population, and all domains are valid components of the overall scores (HR-QoL 0.836, TLQI 0.866).

### Statistical analysis

All analyses were conducted in StataNow 18 BE (Stata Corp LLC, College Station, TX, USA). Disease severity was classified based on flea counts. Pupils with one to ten fleas were classified as mild, while those with more than ten fleas were classified as severe disease based on our previous study in this population [16].

**TLQI** Initially the individual domains were assessed by calculating the number of infected pupils reporting “quite a lot” or “very much” impact (score of 2 or 3) for each domain to identify the most affected domains. Correlation of disease severity with each domain was tested using the Chi^2^ test. The total TLQI score was calculated by summing all eight domains for each pupil. For comparison with other studies, a five-level ordinal variable was generated; no impact (0–1), small (2–5), moderate (6–10), large (11–15) and very large (16–24) based on our previous studies in Kenya [19,21] and the original children’s CDLQI [38] (see S1 Fig for the thresholds in a frequency histogram). Association of the TLQI score with disease severity, levels of pain and itching experienced and other possible explanatory and confounding variables were assessed using a mixed effect negative binomial model on account of the positively skewed distribution of the scores (S1 Fig). The negative binomial model was preferred over Poisson for this count data since it accommodates data for which the mean and variance are not equal. A mixed effect model using the school unique identifier as a random effect was chosen to adjust for unobserved heterogeneity between schools which may have been introduced by the clustered nature of the observations. That is, children were selected from schools, and children within a school may have given similar responses as a result of them being influenced or affected by factors relating to that school, neighborhood or community that was not recorded. Failing to account for this clustering would likely underestimate the model standard errors, leading to an elevated type I error and incorrect conclusions to be drawn regarding significantly associated variables. All explanatory variables were included in a bivariable analysis and those with a p-value less than 0.2 were included in the full multivariable model. Backward elimination was used to develop the final model, removing independent variables with a p-value greater than 0.05 in a stepwise process, one by one, to achieve the lowest Akaike information criteria (AIC) value. Outcomes are presented as adjusted β coefficients with 95% confidence intervals and p-values.

**KIDSCREEN52 (HR-QoL)** The KIDSCREEN scores were processed according to the standard procedures prescribed in the manual [39]. Any negatively coded scale was inverted to be positively coded. The total score for all questions in a domain was calculated. This total domain score was then transformed into a Rasch person parameter and then to a T-value using the international norm values available online [40], Stata code in S1 Text. This transformation aims to obtain a parametric measure with a mean of 50 and a standard deviation of 10 for each domain. The domain T scores were summed up to obtain the HR-QoL score. The lower the score the lower the quality of life. Based on the unusual bimodal distribution of the total HR-QoL scores (S2 Fig), mixed effect generalized linear models with a gaussian distribution and identity link were used to test the association of disease status and severity for each domain individually and for the T-values (HR-QoL) with pain and itching levels experienced, and independent variables. The generalized linear models were chosen since they are more flexible than classical linear models, to accommodate data that is not perfectly normally distributed, as was the case for this dataset. The school unique identifier was used as a random effect to adjust for the clustered nature of the observations. The explanatory variables were included in a bivariable analysis and those with a p-value less than 0.2 were included in the full multivariable model. Backward elimination was used to develop the final model as described above. Outcomes are presented as adjusted β coefficients with 95% confidence intervals and p-values.

#### Explanatory variables.

The selection of possible explanatory and confounding variables to include in multivariable models was guided by a conceptual framework illustrated in S3 Fig. Variables associated with the disease, and/or considered to be associated with quality of life such as parent and family characteristics, parenting style, and pupil characteristics and behavior and had no conceptual overlap with the outcome variable were included. The selection of these variables was conducted first by LE, UF and BO in discussion and reviewed by AA. It did not involve community participation. The variables included were different for the two quality-of-life measures.

#### Missing responses.

All participants were expected to provide responses to all questions (variables). Missing responses in the independent variables were ‘missing at random’ and were all less than 5% of all observations (S3 Table). We therefore performed these analyses under the valid missing at random assumption, as we used likelihood approaches [41]. Final models included no less than 90% of the observations.

## Results

### Pupil participants

**Fig 2:**
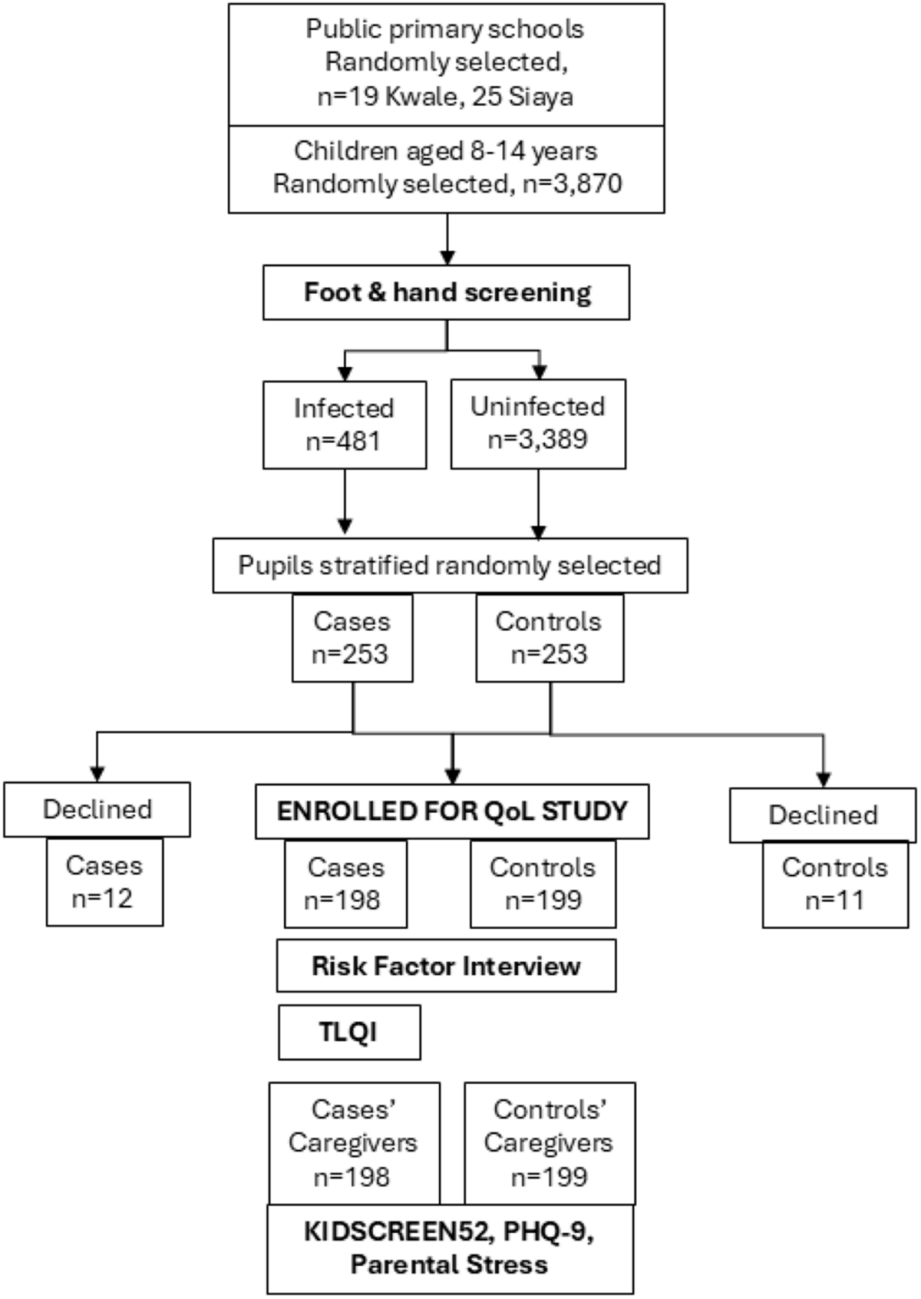
Flow chart of pupils examined for tungiasis, selected, and enrolled for the quality-of-life study.

A total of 3,870 pupils were examined for tungiasis and 481 were found to be infected as described previously [16] and illustrated in the flow chart in Fig 2. From these, 210 infected and 210 uninfected pupils were stratified randomly selected to participate, but only 198 infected and 199 uninfected pupils and their caregivers consented to enroll into the quality-of-life study and were included in analyses (Fig 2). As intended by the selection criteria, they were evenly distributed between Kwale (195) and Siaya (202), and between boys (227) and girls (168). Of the infected pupils, 103 (52.0%) had severe disease (more than 10 fleas). Distribution of participants by all covariables included in the multivariable models are detailed in S3 Table.

### The two instruments

The TLQI was only conducted with 198 infected children. The Proxy-KIDSCREEN52 was conducted with the caregivers of the 198 infected children, plus caregivers of the 199 uninfected children.

### The tungiasis life quality index

With this instrument we assessed the level of impact of tungiasis on the disease specific quality of life of infected pupils and compare pupils with mild disease to those with severe disease. The higher the score the greater the impact and the lower the quality of life.

The percent of pupils who reported experiencing “quite a lot” or “very much” impact was highest for disturbed sleep, feelings of shame and sadness while those domains with the lowest percent of pupils experiencing these levels of impact were friendships and bullying (Table 2).

**Table 2:** Percent of pupils who reported experiencing “quite a lot” or “very much” for each domain of the TLQI by disease severity.

Domain	All cases n = 196 (%)	Mild n = 94 (%)	Severe n = 102 (%)	Chi^2^ p-value
Sleep disturbance	38.3	30.9	45.1	0.040
Feeling ashamed	35.7	28.7	42.2	0.050
Feeling sad	33.7	24.5	42.4	0.009
Concentration in class	29.1	26.6	31.4	0.462
Mobility	27.0	21.3	32.4	0.081
Feeling angry	26.5	19.2	33.3	0.025
Bullying	23.0	21.3	24.5	0.591
Friendships	19.9	11.7	27.5	0.006

A higher percentage of pupils with severe tungiasis reported higher levels of impact than those with mild disease in all domains (p < 0.050) except concentration in school and bullying.

**Fig 3:**
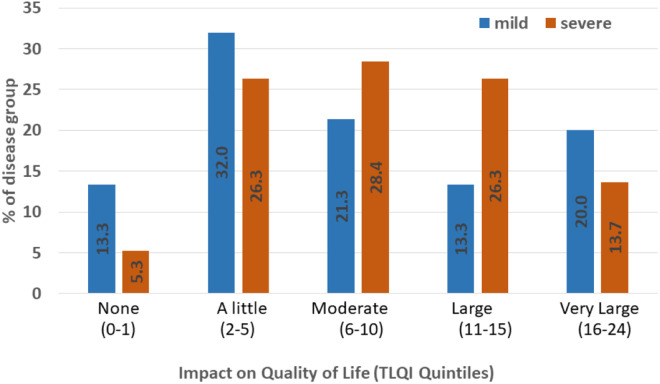
Frequency distribution of pupils across the five TLQI categories reflecting impact on quality of life by disease severity.

When the scores of all domains were combined for each child, the median TLQI score for all pupils was 7 (interquartile range (IQR) 4.0 −12.5). The median score for mild cases was 7 (IQR 3 − 12) and for severe cases was 8 (IQR 5 − 13). When grouped into five categories of impact, of all cases 62.4% reported experiencing a moderate to very large impact on their quality of life. Of the mild cases, 54.7% experienced this level of impact and 68.4% of severe cases (Fig 3). Among the severe cases 31.6% reported none or only a small impact and 20% of mild cases reported a very large impact.

In the bivariable regression analyses for TLQI we found that severe tungiasis was associated with a higher TLQI (β 0.24, 95% CI 0.03 − 0.45, p = 0.027; S4 Table). Once adjusted for other covariables in the final multivariable model this association remained (adjusted β 0.27, 95% CI 0.07 − 0.48, p = 0.009, Table 3).

**Table 3:** Multivariable mixed effect negative binomial linear regression analysis of associations of TLQI with tungiasis severity and covariables. The school unique identifier was used as a random effect.

					Full model (n = 187)		Final model (n = 189)	
Variables	Categories	N^1^	Median	IQR	Adjusted β Coefficient (95% CI^2^)	P^3^	Adjusted β Coefficient (95% CI)	P
Tungiasis severity	Mild	93	7	3 − 10	ref		ref	
	Severe	102	10	4 − 14	0.24 (0.03 − 0.44)	0.027	0.27 (0.07 − 0.48)	0.009
Region	Kwale	95	10	4 − 13	ref			
	Siaya	100	7	3 − 11	-0.07 (-0.34 − 0.20)	0.634		
Sex	Female	56	7	4 − 11	ref			
	Male	139	7	3 − 13	0.04 (-0.18 − 0.26)	0.720		
Pupil age		196			0.02 (-0.04 − 0.08)	0.536		
Adults live with	Both parents	121	7	4 − 13	ref			
	Others	77	7	3 − 13	-0.03 (-0.39 − 0.32)	0.861		
Primary caregiver	Mother	145	7	3 − 13	ref		ref	
	Other adult	52	7	4 − 12	0.37 (-0.03 − 0.76)	0.067	0.38 (0.06 − 0.71)	0.021
Mother’s education	Secondary	68	5	3 − 13	ref			
	None	16	13	6 − 17	0.16 (-0.22 − 0.53)	0.415		
	Primary	87	9	5 − 12	0.00 (-0.29 − 0.29)	0.985		
	Don’t know	27	7	3 − 12	0.22 (-0.15 − 0.59)	0.250		
Father’s education	Secondary	62	5	3 − 12	ref			
	None	11	7	3 − 11	-0.68 (-1.19 − -0.18)	0.008		
	Primary	88	10	5 − 14	-0.11 (-0.40 − 0.17)	0.434		
	Don’t know	36	7	3 − 13	-0.29 (-0.62 − 0.04)	0.089		
Mother away a lot	No	130	8	4 − 13	ref		ref	
	Yes	63	7	2 − 11	-0.37 (-0.70 − -0.04)	0.030	-0.45 (-0.77 − -0.14)	0.005
Parents attend school meetings	Often	83	10	3 − 11	ref		ref	
	Not often	114	6	3 − 11	-0.27 (-0.49 − -0.06)	0.014	-0.30 (-0.50 − -0.11)	0.003
Parents check homework	Never	35	7	3 − 13	ref			
	Sometimes	163	7	4 − 13	-0.08 (-0.36 − 0.20)	0.582		
Family member ill some months	No	117	7	3 − 11	ref			
	Yes	78	10	5 − 15	0.23 (-0.01 − 0.47)	0.059		
Caregiver depressed	No	114	6	3 − 10	ref		ref	
	Yes	81	11	5 − 16	0.31 (0.10 − 0.53)	0.005	0.28 (0.08 − 0.49)	0.006
Caregiver time spend with child	A little	146	8	4 − 13	ref			
	A lot	47	6	3 − 13	-0.10 (-0.35 − 0.16)	0.466		
Child fears a family member	No	130	7	3 − 12	ref			
	Yes	65	10	5 − 14	0.01 (-0.24 − 0.26)	0.913		
Caregiver hugs child	No	139	8	5 − 14	ref		ref	
	Yes	55	4	2 − 10	-0.37 (-0.64 − -0.10)	0.007	-0.45 (-0.70 − -0.20)	<0.001
Discipline style	Other	62	7	3 − 11	ref			
	Beaten	136	8	4 − 13	0.03 (-0.20 − 0.26)	0.788		
HHH^4^ sex	Female	42	7	3 − 12	ref			
	Male	151	7	4 − 13	0.26 (-0.09 − 0.61)	0.143		
HHH age		196			0.00 (-0.01 − 0.01)	0.638		
Caregiver sex	Female	169	7	4 − 13	ref			
	Male	24	7	3 − 12	0.00 (-0.37 − 0.37)	0.990		
Caregiver age		196			0.00 (-0.01 − 0.02)	0.648		

^1^number, ^2^confidence interval, ^3^ p-value, ^4^head of household.

In the final multivariable model other covariables were also associated with the TLQI. Children whose caregivers were classified as depressed had a higher TLQI (adjusted β 0.28, 95% CI 0.08 − 0.49, p = 0.006) than those whose caregiver was not depressed, which means they experienced a higher impact from tungiasis than those whose caregivers were not depressed (Table 3). Other factors associated with a higher TLQI included if a child’s main caregiver was not their mother (adjusted β 0.38, 95% CI 0.06 − 0.71, p = 0.021). Conversely, children whose caregiver reported they hug and cuddle their child had a lower TLQI than children whose caregiver did not, which means they experienced a lower impact of tungiasis (adjusted β -0.45, 95% CI -0.70 − -0.20, p < 0.001). Additionally, parents who only attended school meetings sometimes vs. often (adjusted β -0.30, 95% CI -0.50 − -0.11, p = 0.003), and if a child’s mother was away a lot (adjusted β -0.45, 95% CI -0.77 − -0.14, p = 0.005) were associated with lower TLQI scores (Table 3).

### General health related quality of life assessed by the caregiver (KIDscreen52)

With this instrument we compared the scores of uninfected pupils with pupils having mild and severe tungiasis separately. The lower the score the lower the health-related quality of life.

The domains with lowest mean scores for all pupil groups were financial resources, social acceptance, and physical well-being (Table 4). The financial resources were extremely low irrespective of disease status. Compared to uninfected children, those with severe tungiasis were associated with lower scores in the domains of: psychological well-being (β -3.53, 95% CI -6.74 − -0.32, p = 0.031), moods and emotions (β -4.16, 95% CI -7.05 − -1.27, p = 0.005), self-perception (β -4.35, 95% CI -6.99 − -1.71, p = 0.001), school environment (β -3.05, 95% CI -5.88 − -0.22, p = 0.034) and social acceptance (β-5.86, 95% CI-9.51 − -2.21, p = 0.002) (Table 4).

**Table 4:** Bivariable mixed effect linear regression analysis of the association of individual domains of HR-QoL (KIDSCREEN52) with tungiasis, using the school unique identifier as a random effect.

	Disease group	Mean	SD^1^	β Coefficient	95% CI^2^		P
Physical well-being	Uninfected	51.9	12.6				
	Mild	53.8	13.7	1.90	-1.27	5.07	0.240
	Severe	49.0	13.2	-2.83	-5.91	0.26	0.073
Psychological well-being	Uninfected	52.0	13.0				
	Mild	53.6	14.1	1.67	-1.63	4.97	0.321
	Severe	48.4	14.1	-3.53	-6.74	-0.32	0.031
Moods & emotions	Uninfected	59.9	11.2				
	Mild	58.3	13.4	-1.65	-4.62	1.31	0.275
	Severe	55.8	12.9	-4.16	-7.05	-1.27	0.005
Self-perception	Uninfected	54.7	10.9				
	Mild	55.0	11.4	0.34	-2.37	3.05	0.806
	Severe	50.3	11.3	-4.35	-6.99	-1.71	0.001
Autonomy	Uninfected	48.3	13.4				
	Mild	48.9	14.1	0.60	-2.65	3.84	0.719
	Severe	48.5	12.4	0.22	-2.94	3.38	0.892
Parent relations	Uninfected	53.8	12.0				
	Mild	55.8	12.5	2.00	-1.00	5.00	0.191
	Severe	52.6	12.7	-1.20	-4.12	1.72	0.422
Financial Resources	Uninfected	28.1	7.3				
	Mild	27.8	8.9	-0.31	-2.24	1.61	0.75
	Severe	27.6	8.0	-0.48	-2.35	1.40	0.618
Social support & peers	Uninfected	46.7	14.0				
	Mild	50.6	15.2	3.99	0.52	7.45	0.024
	Severe	48.9	13.8	2.20	-1.17	5.57	0.201
School Environment	Uninfected	58.4	11.5				
	Mild	60.3	12.1	1.91	-1.00	4.81	0.198
	Severe	55.3	12.6	-3.05	-5.88	-0.22	0.034
Social Acceptance	Uninfected	42.7	13.9				
	Mild	41.4	17.5	-1.26	-5.01	2.49	0.510
	Severe	36.8	16.1	-5.86	-9.51	-2.21	0.002

^**1**^ standard deviation, ^2^confidence interval.

When all the domain scores were combined into the overall HR-QoL score for each child, the mean scores were lowest for children with severe tungiasis at 473.4 (sd 82.0) compared to 496.5 (sd 77.8) for uninfected children (Table 5). In bivariable analysis there was no association with infection when mild and severe cases were combined (β -7.93, 95% CI -23.16 − 7.29, p = 0.307), but when cases were separated by disease severity, severe disease was associated with a lower HR-QoL relative to no infection (β -21.15, 95% CI -39.63 − -2.68, p = 0.025, S5 Table). There was no association of the HR-QoL with mild tungiasis relative to uninfected children. In the multivariable model adjusting for covariables, the association between HR-QoL and severe tungiasis was no longer statistically significant (adjusted β -13.28, 95% CI -30.71 − 4.15, p = 0.135, Table 5).

**Table 5:** Multivariable mixed effect linear regression analysis of associations of disease status and covariables with HR-QoL (KIDSCREEN52) using the school unique identifier as a random effect.

					Full multivariable (n = 361)		Final multivariable (n = 376)	
Variable	Categories	N^1^	mean	sd^2^	Adjusted β Coefficient (95% CI^3^)	P^4^	Adjusted β Coefficient (95% CI)	P
Tungiasis status	Uninfected	199	496.5	77.8	ref		ref	
	Mild	95	505.6	85.9	9.75 (-9.00 − 28.50)	0.308	9.29 (-8.59 − 27.17)	0.309
	Severe	103	473.4	82	-10.09 (-29.37 − 9.18)	0.305	-13.28 (-30.71 − 4.15)	0.135
Region	Kwale	195	475.1	62.9	ref		ref	
	Siaya	202	509.6	93.3	35.89 (18.18 − 53.60)	<0.001	38.62 (21.83 − 55.41)	<0.001
Sex	Girls	168	495.5	81.9	ref			
	Boys	227	489.9	81.4	-3.48 (-18.83 − 11.88)	0.657		
Pupil age		391			-1.32 (-5.51 − 2.88)	0.538		
Adults child lives with	Both parents	252	491.4	79.1	ref			
	Others	143	493.8	85.9	6.96 (-19.71 − 33.63)	0.609		
Relationship to caregiver	Parent	295	489.4	80.6	ref			
	Other	99	501.6	83.9	3.66 (-26.38 − 33.69)	0.811		
Who child chooses to go to when unwell	Mother	191	507.4	79.9	ref			
	Others	199	477.9	81.1	-32.26 (-48.84 − -15.68)	<0.001	-26.19 (-41.13 − -11.24)	0.001
Orphaned	No	371	493.3	80.3	ref			
	Yes	26	483	98.7	-3.48 (-39.46 − 32.50)	0.850		
Family ill months	No	241	511.2	86.6	ref		ref	
	Yes	154	462.7	62.6	-28.89 (-45.09 − -12.68)	<0.001	-26.18 (-41.63 − -10.74)	0.001
Family disability	No	365	494	81.4	ref			
	Yes	28	475.9	82.3	-1.00 (-31.99-30.00)	0.950		
Mother away a lot	No	274	483.9	80.7	ref		ref	
	Yes	117	510.9	81.7	11.09 (-10.88 − 33.07)	0.322	20.30 (3.47 − 37.14)	0.018
Father away a lot	No	121	479.8	77.3	ref		ref	
	Yes	263	500.9	81.7	27.32 (9.27 − 45.37)	0.003	29.48 (12.46 − 46.50)	0.001
Caregiver depressed	No	255	508.4	79.1	ref		ref	
	Yes	142	464.4	78.6	-40.58 (-56.63 − -24.52)	<0.001	-40.34 (-55.91 − -24.76)	<0.001
HHH5 sex	Female	87	487.2	83.4	ref			
	Male	302	494.2	81.5	8.07 (-15.22 − 31.36)	0.497		
HHH age		389			-0.30 (-1.12 − 0.53)	0.481		
Caregiver sex	Female	355	491.3	81.3	ref			
	Male	34	507	87.8	-4.61 (-33.97 − 24.75)	0.758		
Caregiver age		389			0.87 (-0.13 − 1.86)	0.087		
Mother education	None	38	471.9	76.3	ref			
	Don’t know	159	493.2	87.8	-15.16 (-46.48 − 16.17)	0.343		
	Primary	145	485.1	70.7	-11.04 (-38.85 − 16.78)	0.437		
	Secondary	53	523.6	86.6	9.90 (-24.93 − 44.74)	0.577		
Father education	None	13	474.3	76	ref			
	Don’t know	176	486.8	85.6	-7.59 (-52.54 − 37.36)	0.741		
	Primary	125	489.3	75.5	0.99 (-42.92 − 44.89)	0.965		
	Secondary	79	514.1	80	9.98 (-34.51 − 54.48)	0.660		

^1^number, ^2^standard deviation, ^3^confidence interval, ^4^ p-value, ^5^head of household.

Variables that were associated with a higher HR-QoL included: residing in Siaya county (adjusted β 38.62, 95% CI 21.83 − 55.41, p < 001); if the father was away a lot (adjusted β 29.48, 95% CI 12.46 − 46.50, p = 0.001) and if the mother was away a lot (adjusted β 20.30, 95% CI 3.47 − 37.14, p = 0.018) (Table 5). Variables associated with a lower HR-QoL included: if the caregiver was classified as depressed (adjusted β -40.34, 95%CI -55.91− −24.76, p < 0.001), if a family member was chronically ill (adjusted β - 26.18, 95% CI -41.63 − -10.74, p < 0.001,) and if the child chooses to go to an adult other than their parent when feeling unwell (adjusted β 26.19, 95% CI -41.13 − -11.24, p = 0.001)(Table 5).

### Pain and itching

Two of the major symptoms of tungiasis are pain and itching caused by the inflammation induced by embedded fleas [14]. To explore whether pain and itching levels might be associated with the quality-of-life measures, each infected child was asked about their experience of pain and itching in their feet as part of the interview for the TLQI. In bivariable regression analysis the higher the category of pain the higher the TLQI (β 0.36, 95% CI 0.27 − 0.45, p = 0.001) and likewise for itching (β 0.35, 95% CI 0.26 − 0.45, p = 0.001). There was no significant association of pain and itching with the HR-QoL assessment conducted by their caregiver (Table 6).

**Table 6:** Association of pain and itching with quality-of-life scores for all infected children (n = 198).

TLQI^1^	β Coefficient^2^	95% CI^3^		P
pain	0.36	0.27	0.45	<0.001
itch	0.35	0.26	0.45	<0.001
**HR-QoL** ^**4**^				
pain	-6.59	-18.01	4.83	0.258
itch	-8.11	-19.62	3.40	0.167

^1^Tungiasis quality of life index.

^2^β coefficient from mixed effect linear regression model with school ID as random effect.

^3^confidence interval.

^4^Health related quality of life score (KIDSCREEN52).

## Discussion

In this study we set out to determine whether tungiasis had an impact on children’s quality of life and whether there was any difference between mild and severe disease, using a disease-specific, and a proxy general-health related instrument. This study reinforced previous findings that tungiasis, particularly severe disease, impacts children’s quality of life considerably and identified that children feel the most impact on their sleep and tungiasis makes them feel ashamed and sad. We also aimed to determine whether any other factors in a child’s life may also be associated with quality of life and possibly affect the relationship between tungiasis and quality of life. The most important new finding is that caregiver depression was strongly associated with both measures (tungiasis-specific and general health) of quality of life. Children whose caregivers were classified as depressed had a higher TLQI and lower proxy HR-QoL. Caregiver depression has previously been associated with poor physical and cognitive development and life satisfaction among children [42,43]. A depressed caregiver gives less attention to their children [44], including their children’s personal hygiene [45]. Low frequency of bathing and soap-use by children have been shown to be risk factors for tungiasis [9,12]. Consequently, not only could caregiver depression put children at greater risk for infections such as tungiasis (not assessed here), but also to being more vulnerable to the impact of disease on their quality of life, as seen here. Further studies are needed to confirm this, but in the meantime interventions for tungiasis and those aiming to improve children’s quality of life should incorporate a component on caregiver mental health.

An important component of parenting is the display of affection through physical contact and hugging the child for their psychological well-being and development, absence of which can have a lifelong impact on the child [46]. In the present study, if a caregiver reported that they currently hug the child, their child had a lower TLQI; tungiasis had a lower impact on their quality of life. Another variable reflecting parent attention and care for the child, “attending school meetings”, was also negatively associated with the TLQI, that is, more parent attention in a child’s life was associated with tungiasis having a lower impact on the child. These findings suggest that a caregiver can reduce the impact tungiasis has on their child by changing their parenting style even if they cannot eliminate the disease. This should be investigated further using validated instruments for parenting styles along with developing an intervention incorporating parenting styles.

The proxy HR-QoL did not include a sleep disturbance domain, but this was the most affected domain in the TLQI. Sleep deprivation is a common symptom of SNTDs; tungiasis [19,21], cutaneous larva migrans [47] and scabies [48] which is concerning since it impacts cognitive performance [49], learning and memory formation [50], and can lead to depression, other psychological disorders and behavioral changes [51]. In fact, assessments done with these same children in this study showed that tungiasis was associated with significant cognitive impacts [20]. Further studies with a larger sample size are needed to confirm the associations of tungiasis with poor maternal and child mental health and cognitive development and to explore possible causal pathways.

There was considerable discrepancy in the responses to the social acceptance (bullying) domain in the two instruments. It was the most affected domain according to caregivers, while it was the least affected domain according to the children themselves. Past studies have found it is quite common for there to be low correlation between the child and caregiver assessments [52,53]. Children themselves may not want to admit to a stranger that they are being bullied and excluded, while their caregivers are aware of the situation and did not have the same inhibitions. Alternatively, the caregiver may think that the child is being bullied when in fact they were not. Poor correlation between caregiver and child assessments may also be due to poor parent-child relationships; the caregiver not having much knowledge of the child; circumstances and denial on the part of either party; and the pressure on each to give socially acceptable responses and the caregiver’s stress levels [54]. The issue of bullying and social exclusion in tungiasis needs to be investigated further but social exclusion is a common feature reported by adults with NTDs due to the visibility of the disfigurement caused by the disease and community fear of infection and rejection of those affected [3].

The median TLQI score (7, IQR 4 − 12.5) for all patients and the proportion of children who reported a moderate to very high impact (62.4%) in the current study was similar to that seen in the recent nine-county survey in Kenya (9, IQR 4 − 13 and 68.5%) [19]. However, these were considerably lower than the previous study in Kilifi County, Kenya (78%) [21], and those with cutaneous larva migrans in Brazil (71%) [47]. The lower levels of impact may be purely due to the tools having a different number of domains (eight and six respectively) and grouping the scores differently for the quintiles. However, this difference could also be due to cultural differences between the study populations. This suggests that there is need for a standardized instrument and analysis processes to be used for tungiasis studies and perhaps all parasitic skin diseases. The good internal consistency and frequency of a higher rating among children with severe disease, suggests the addition of the two extra variables, feelings of anger and sadness were probably appropriate, but the whole instrument requires systematic validation.

What is unexpected in this study is that 31.6% of children with severe disease reported little or no impact and 20% of children with mild disease reported high levels of impact. Kehr et al [55] suggest the high level of keratinization of the feet associated with fleas in chronic disease means some children may experience less impact. Another possible explanation is that children’s responses are influenced by their past experience, familial, cultural and cognitive factors that have been shown to play a role in pain perception and expression [56].

Previous qualitative studies on the impact of skin-related neglected tropical diseases such as lymphatic filariasis, cutaneous leishmaniasis and leprosy, on quality of life have found females experienced more impact than males [57,58]. In our current study there was no such association between sex and either quality of life measure. This may well be because our study was of children aged 8–14 years whereas most previous studies have focused on adults who have specific gender roles in many cultures which seem to be impacted differently. For instance, women with skin NTDs find it more difficult to get married, to participate in social events and attend to any activity which involves public places.

The TLQI was positively associated with levels of pain and itching which is perhaps not unexpected since higher levels of pain are likely to make it more difficult to walk and sleep. The question relating to concentration in class is specifically asked in relation to the itching caused by the fleas, and itching in other chronic skin diseases has been shown to worsen at night, causing sleep disturbance [59,60]. The lack of association of the children’s pain and itching levels with the HR-QoL is perhaps a reflection of the fact that it was a proxy assessment of the child’s quality of life and that pain and itching are not associated with its various domains when assessed by the caregiver rather than the child. This result combined with the finding that severe disease was not associated with the HR-QoL suggests it is not a suitable instrument to use to assess the impact of tungiasis in a child’s quality of life.

### Study limitations

One possible limitation of the study was that while there was some conceptual overlap, the two instruments chosen for the study were quite different from each other, one being disease-specific, implemented with affected children while the other addressed general health and was implemented with the caregiver. Ideally, we would have included the KIDSCREEN52 self-assessment instrument for the child’s general HR-QoL. However, we did not wish to overburden the child participants who were also completing extensive cognitive assessments at the same visit [20], so the proxy instrument was used instead. The good internal consistency (Cronbach’s alpha >0.8) in both instruments suggests they reliably measured the tungiasis- or health-related quality of life respectively, in this study population. The good internal consistency of the HR-QoL also indicates the international norm values for the Rasch person parameters used for the KIDSCREEN52 transformations were suitable for this population. At the domain level both instruments found children with severe tungiasis experienced more sadness/depressive moods and shame/low self-esteem than children with mild tungiasis (less than 10 fleas).

Another limitation related to the instruments was that the wording of the questions in the TLQI was ambiguous, particularly that relating to friendships. It is not clear whether the responses from the children indicate a positive or negative impact on their friendships. In this study the instrument appears to be suitable, but it could still be improved and needs systematic validation.

## Conclusions

The cross-sectional study design meant that we have been able to demonstrate an association of severe tungiasis with low disease-specific quality of life. and that other factors were also associated with both disease-specific and general health related quality of life. Further studies are needed to investigate the causality of these associations. The lack of association of tungiasis with the HR-QoL as assessed by the proxy-KIDSCREEN52 instrument suggests this may not be an appropriate instrument to assess the impact of tungiasis on quality of life, but the individual KIDSCREEN instrument may be better. That caregiver mental health and parenting style, have now been shown to be associated with both disease incidence and quality of life of children in these resource poor communities highlights the need to incorporate psychosocial and mental health activities into intervention programs to reduce the incidence of disease as well as its impact. While WHO’s Road Map [1] and Framework [2] for the elimination of NTDs encourage the integration of mental health services to reduce the impact of NTDs, it appears mental health services also need to be part of prevention programs to reduce the transmission of some of these diseases.

## Supporting information

S1 Table Tungiasis Life Quality Index (TLQI) questionnaire. (DOCX)

S2 Table KIDSCREEN52 Questionnaire. (DOCX)

S1 Fig Frequency histogram of TLQI scores for all patients. (DOCX)

S1 Text Stata code for transforming KIDSREEN52 scores. (DOCX)

S2 Fig Frequency histogram of HR-QOL T-values for all patients. (DOCX)

S3 Fig Conceptual framework for the association of tungiasis with Quality of Life. (DOCX)

S3 Table Participant Distribution by dependent and independent variables and disease status. (DOCX)

S4 Table Bivariable mixed effect negative binomial regression analysis for TLQI. (DOCX)

S5 Table Bivariable mixed effect linear regression analysis for HR-QoL. (DOCX)

S1 Checklist STROBE Checklist. [61].
(DOCX)
