# Supplementary material for: Assessing the impact of tungiasis on children’s quality of life in Kenya
Source: PLoS Negl Trop Dis. 2025 Sep 8;19(9):e0012606. doi: 10.1371/journal.pntd.0012606 (PMC12431661; doi:10.1371/journal.pntd.0012606)
Supplement: S1 Table — (DOCX) [file pntd.0012606.s001.docx]

# S1_Table:

# Tungiasis Life Quality Index (TLQI) questionnaire

**Title:** **Assessing the impact of tungiasis on children’s quality of life in Kenya.**

**Journal:** Quality of Life Research

**Author names:**

Lynne Elson^1,2, *^, Berrick Otieno^1^, Abneel K Matharu^3,4^, Naomi Rithi^3^, Esther Chongwo^5^, Francis Mutebi^6^, Hermann Feldmeier^7^, Jürgen Krücken^4^, Ulrike Fillinger^3,5^, Amina Abubakar^1,5^

**Affiliations:**

^1^ Kenya Medical Research Institute (KEMRI)-Wellcome Trust, Kilifi, Kenya. Orcid ID: 0000-0003-2264-4459.

^2^ Centre for Tropical Medicine and Global Health, Nuffield Department of Medicine, University of Oxford, United Kingdom.

^3^ International Centre of Insect Physiology and Ecology, Mbita, Kenya

^4^ Institute for Parasitology and Tropical Veterinary Medicine, Freie Universität Berlin, Germany

^5^Institute for Human Development, Aga Khan University, Nairobi, Kenya

^6^ School of Veterinary Medicine and Animal Resources, College of Veterinary Medicine, Animal Resources and Biosecurity, Makerere University, Kampala, Uganda

^7^ Institute of Microbiology, Infectious Diseases and Immunology, Charité University Medicine, Berlin, Germany

**Corresponding Author:**

Lynne Elson

Kenya Medical Research Institute (KEMRI)-Wellcome Trust, Hospital Road, Kilifi, Kenya

Email: lynne.elson@gmail.com

## S1_Table. Tungiasis Life Quality Index (TLQI) questionnaire

|  | Variable/ Question | categories |
| --- | --- | --- |
| 1 | Region | radio, Required   \| s \| SI \| \| --- \| --- \| \| k \| KW \| |
| 2 | child id | text, Required, Identifier |
| 3 | child name | text, Required, Identifier |
| 4 | school id | text |
|  | ***During the last week rate the following according to the scales*** |  |
| 5 | How embarrassed or ashamed did you feel because of the jiggers? | radio (Matrix), Required   \| 0 \| Not at all \| \| --- \| --- \| \| 1 \| Only a little \| \| 2 \| Quite a lot \| \| 3 \| Very much \| |
| 6 | How much do the jiggers make it difficult for you to walk/run? | radio (Matrix), Required   \| 0 \| Not at all \| \| --- \| --- \| \| 1 \| Only a little \| \| 2 \| Quite a lot \| \| 3 \| Very much \| |
| 7 | How much do the jiggers affect your concentration in class because of the itching? | radio (Matrix), Required   \| 0 \| Not at all \| \| --- \| --- \| \| 1 \| Only a little \| \| 2 \| Quite a lot \| \| 3 \| Very much \| |
| 8 | How much do the jiggers affect your sleep? | radio (Matrix), Required   \| 0 \| Not at all \| \| --- \| --- \| \| 1 \| Only a little \| \| 2 \| Quite a lot \| \| 3 \| Very much \| |
| 9 | How much do the jiggers affect your friendships? | radio (Matrix), Required   \| 0 \| Not at all \| \| --- \| --- \| \| 1 \| Only a little \| \| 2 \| Quite a lot \| \| 3 \| Very much \| |
| 10 | How much are other children mean/cruel/unkind to you? | radio (Matrix), Required   \| 0 \| Not at all \| \| --- \| --- \| \| 1 \| Only a little \| \| 2 \| Quite a lot \| \| 3 \| Very much \| |
| 11 | How much do the jiggers make you feel sad? | radio (Matrix), Required   \| 0 \| Not at all \| \| --- \| --- \| \| 1 \| Only a little \| \| 2 \| Quite a lot \| \| 3 \| Very much \| |
| 12 | How often do you get angry? | radio (Matrix), Required   \| 0 \| Not at all \| \| --- \| --- \| \| 1 \| Only a little \| \| 2 \| Quite a lot \| \| 3 \| Very much \| |
| 13 | How much pain do you feel? | radio (Matrix), Required   \| 0 \| Not at all \| \| --- \| --- \| \| 1 \| Only a little \| \| 2 \| Quite a lot \| \| 3 \| Very much \| |
| 14 | How much Itching do you feel? | radio (Matrix), Required   \| 0 \| Not at all \| \| --- \| --- \| \| 1 \| Only a little \| \| 2 \| Quite a lot \| \| 3 \| Very much \| |
| 15 | Form Status Complete? | dropdown   \| 0 \| Incomplete \| \| --- \| --- \| \| 1 \| Unverified \| \| 2 \| Complete \| |
