# Supplementary material for: Assessing the impact of tungiasis on children’s quality of life in Kenya
Source: PLoS Negl Trop Dis. 2025 Sep 8;19(9):e0012606. doi: 10.1371/journal.pntd.0012606 (PMC12431661; doi:10.1371/journal.pntd.0012606)
Supplement: S2 Table — (DOCX) [file pntd.0012606.s002.docx]

# S2_Table: KIDSCREEN52 Questionnaire

**Title:** **Assessing the impact of tungiasis on children’s quality of life in Kenya.**

**Journal:** Quality of Life Research

**Author names:**

Lynne Elson^1,2, *^, Berrick Otieno^1^, Abneel K Matharu^3,4^, Naomi Rithi^3^, Esther Chongwo^5^, Francis Mutebi^6^, Hermann Feldmeier^7^, Jürgen Krücken^4^, Ulrike Fillinger^3,5^, Amina Abubakar^1,5^

**Affiliations:**

^1^ Kenya Medical Research Institute (KEMRI)-Wellcome Trust, Kilifi, Kenya. Orcid ID: 0000-0003-2264-4459.

^2^ Centre for Tropical Medicine and Global Health, Nuffield Department of Medicine, University of Oxford, United Kingdom.

^3^ International Centre of Insect Physiology and Ecology, Mbita, Kenya

^4^ Institute for Parasitology and Tropical Veterinary Medicine, Freie Universität Berlin, Germany

^5^Institute for Human Development, Aga Khan University, Nairobi, Kenya

^6^ School of Veterinary Medicine and Animal Resources, College of Veterinary Medicine, Animal Resources and Biosecurity, Makerere University, Kampala, Uganda

^7^ Institute of Microbiology, Infectious Diseases and Immunology, Charité University Medicine, Berlin, Germany

**Corresponding Author:**

Lynne Elson

Kenya Medical Research Institute (KEMRI)-Wellcome Trust, Hospital Road, Kilifi, Kenya

Email: [lynne.elson@gmail.com](mailto:lynne.elson@gmail.com)

## S2_Table: KIDSCREEN52 Questionnaire

| **KIDSCREEN52 PARENTAL ASSESSMENT OF CHILD QUALITY OF LIFE** | | | |
| --- | --- | --- | --- |
| 468 | [dt_cs] | Date | text (date_ymd) |
| 469 | [region_cld_score] | Region | radio, Required   \| s \| SI \| \| --- \| --- \| \| k \| KW \| |
| 470 | [sc_id_cs] | School id: | text (number) |
| 471 | [in_cld_id_cs] | Index child id | text (number) |
| 472 | [childs_health] | Section Header: *KIDSCREEN PARENTAL ASSESSMENT OF CHILD QUALITY OF LIFE How is your child? How does she/he feel? This is what we would like to know from you. Please answer the following questions to the best of your knowledge, ensuring that the answers you give reflect the perspective of your child. Please try to remember your child's experiences over the last week..*  In general, how would your child rate her/his health? | radio   \| 1 \| Extremely good \| \| --- \| --- \| \| 2 \| Very good \| \| 3 \| good \| \| 4 \| fair \| \| 5 \| bad \| |
| 473 | [child_fit_well] | Has your child felt fit and well? | radio, Required   \| 1 \| Not at all \| \| --- \| --- \| \| 2 \| Slightly \| \| 3 \| Moderately \| \| 4 \| Very \| \| 5 \| Very much/ Extremely \| |
| 474 | [child_physically_active] | Has your child been physically active (e.g. running, climbing, biking)? | radio, Required   \| 1 \| Not at all \| \| --- \| --- \| \| 2 \| Slightly \| \| 3 \| Moderately \| \| 4 \| Very \| \| 5 \| Very much/ Extremely \| |
| 475 | [child_run_well] | Has your child been able to run well? | radio, Required   \| 1 \| Not at all \| \| --- \| --- \| \| 2 \| Slightly \| \| 3 \| Moderately \| \| 4 \| Very \| \| 5 \| Very much/ Extremely \| |
| 476 | [child_felt_life_enjoyable] | Section Header: *Psychological well-being Feelings*  Has your child felt that life was enjoyable? | radio, Required   \| 1 \| Not at all \| \| --- \| --- \| \| 2 \| Slightly \| \| 3 \| Moderately \| \| 4 \| Very \| \| 5 \| Very much/ Extremely \| |
| 477 | [child_felt_pleased_alive] | Has your child felt pleased that he/she is alive? | radio, Required   \| 1 \| Not at all \| \| --- \| --- \| \| 2 \| Slightly \| \| 3 \| Moderately \| \| 4 \| Very \| \| 5 \| Very much/ Extremely \| |
| 478 | [child_satisfied_with_life] | Has your child felt satisfied with his/her life? | radio, Required   \| 1 \| Not at all \| \| --- \| --- \| \| 2 \| Slightly \| \| 3 \| Moderately \| \| 4 \| Very \| \| 5 \| Very much/ Extremely \| |
| 479 | [child_in_good_moods] | Has your child been in a good mood? | radio, Required   \| 1 \| Not at all \| \| --- \| --- \| \| 2 \| Slightly \| \| 3 \| Moderately \| \| 4 \| Very \| \| 5 \| Very much/ Extremely \| |
| 480 | [child_been_cheerful] | Has your child felt cheerful? | radio, Required   \| 1 \| Not at all \| \| --- \| --- \| \| 2 \| Slightly \| \| 3 \| Moderately \| \| 4 \| Very \| \| 5 \| Very much/ Extremely \| |
| 481 | [child_had_fun] | Has your child had fun? | radio, Required   \| 1 \| Not at all \| \| --- \| --- \| \| 2 \| Slightly \| \| 3 \| Moderately \| \| 4 \| Very \| \| 5 \| Very much/ Extremely \| |
| 482 | [child_feels_bad_doing_things] | Section Header: *Moods and emotion General Mood*  Has your child felt that he/she does everything badly | radio, Required   \| 1 \| Not at all \| \| --- \| --- \| \| 2 \| Slightly \| \| 3 \| Moderately \| \| 4 \| Very \| \| 5 \| Very much/ Extremely \| |
| 483 | [child_felt_sad] | Has your child felt sad? | radio, Required   \| 1 \| Not at all \| \| --- \| --- \| \| 2 \| Slightly \| \| 3 \| Moderately \| \| 4 \| Very \| \| 5 \| Very much/ Extremely \| |
| 484 | [child_felt_bad_dint_work] | Has your child felt so bad that he/she didn't want to do anything? | radio, Required   \| 1 \| Not at all \| \| --- \| --- \| \| 2 \| Slightly \| \| 3 \| Moderately \| \| 4 \| Very \| \| 5 \| Very much/ Extremely \| |
| 485 | [child_feels_everything_wrong] | Has your child felt that everything in his/her life goes wrong? | radio, Required   \| 1 \| Never \| \| --- \| --- \| \| 2 \| Seldom \| \| 3 \| Quite often \| \| 4 \| Very often \| \| 5 \| Always \| |
| 486 | [child_fed_up] | Has your child felt fed up? | radio, Required   \| 1 \| Never \| \| --- \| --- \| \| 2 \| Seldom \| \| 3 \| Quite often \| \| 4 \| Very often \| \| 5 \| Always \| |
| 487 | [child_felt_lonely] | Has your child felt lonely? | radio, Required   \| 1 \| Never \| \| --- \| --- \| \| 2 \| Seldom \| \| 3 \| Quite often \| \| 4 \| Very often \| \| 5 \| Always \| |
| 488 | [child_felt_under_pressure] | Has your child felt under pressure? | radio, Required   \| 1 \| Never \| \| --- \| --- \| \| 2 \| Seldom \| \| 3 \| Quite often \| \| 4 \| Very often \| \| 5 \| Always \| |
| 489 | [child_happy_way_they_are] | Section Header: *Self-perception Your Child's Feelings*  Has your child been happy with the way he/she is? | radio, Required   \| 1 \| Never \| \| --- \| --- \| \| 2 \| Seldom \| \| 3 \| Quite often \| \| 4 \| Very often \| \| 5 \| Always \| |
| 490 | [child_happy_with_clothes] | Has your child been happy with his/her clothes? | radio, Required   \| 1 \| Never \| \| --- \| --- \| \| 2 \| Seldom \| \| 3 \| Quite often \| \| 4 \| Very often \| \| 5 \| Always \| |
| 491 | [child_worried_with_looks] | Has your child been worried about the way he/she looks? | radio, Required   \| 1 \| Never \| \| --- \| --- \| \| 2 \| Seldom \| \| 3 \| Quite often \| \| 4 \| Very often \| \| 5 \| Always \| |
| 492 | [child_jealous_of_others_looks] | Has your child felt jealous of the way other girls and boys look | radio, Required   \| 1 \| Not at all \| \| --- \| --- \| \| 2 \| Slightly \| \| 3 \| Moderately \| \| 4 \| Very \| \| 5 \| Very much/ Extremely \| |
| 493 | [child_wanted_to_change_body] | Has your child wanted to change something about his/her body | radio, Required   \| 1 \| Never \| \| --- \| --- \| \| 2 \| Seldom \| \| 3 \| Quite often \| \| 4 \| Very often \| \| 5 \| Always \| |
| 494 | [child_had_enough_time_for_themselves] | Section Header: *Autonomy Free Time*  Has your child had enough time for him/herself? | radio, Required   \| 1 \| Never \| \| --- \| --- \| \| 2 \| Seldom \| \| 3 \| Quite often \| \| 4 \| Very often \| \| 5 \| Always \| |
| 495 | [child_do_what_they_want] | Has your child been able to do the things that he/she wants to do in his/her free time? | radio, Required   \| 1 \| Never \| \| --- \| --- \| \| 2 \| Seldom \| \| 3 \| Quite often \| \| 4 \| Very often \| \| 5 \| Always \| |
| 496 | [child_had_enough_time_to_be_outside] | Has your child had enough opportunity to be outside? | radio, Required   \| 1 \| Never \| \| --- \| --- \| \| 2 \| Seldom \| \| 3 \| Quite often \| \| 4 \| Very often \| \| 5 \| Always \| |
| 497 | [child_had_enough_time_meet_friends] | Has your child had enough time to meet friends? | radio, Required   \| 1 \| Never \| \| --- \| --- \| \| 2 \| Seldom \| \| 3 \| Quite often \| \| 4 \| Very often \| \| 5 \| Always \| |
| 498 | [child_had_time_choose_what_to_do_free_time] | Has your child been able to choose what to do in his/her free time? | radio, Required   \| 1 \| Never \| \| --- \| --- \| \| 2 \| Seldom \| \| 3 \| Quite often \| \| 4 \| Very often \| \| 5 \| Always \| |
| 499 | [child_felt_understood_by_parent] | Section Header: *Parent relations and home life Family and Home Life*  Has your child felt understood by his/her parent(s)? | radio, Required   \| 1 \| Not at all \| \| --- \| --- \| \| 2 \| Slightly \| \| 3 \| Moderately \| \| 4 \| Very \| \| 5 \| Very much/ Extremely \| |
| 500 | [child_felt_loved_by_parents] | Has your child felt loved by his/her parent(s)? | radio, Required   \| 1 \| Not at all \| \| --- \| --- \| \| 2 \| Slightly \| \| 3 \| Moderately \| \| 4 \| Very \| \| 5 \| Very much/ Extremely \| |
| 501 | [child_happy_at_home] | Has your child been happy at home? | radio, Required   \| 1 \| Never \| \| --- \| --- \| \| 2 \| Seldom \| \| 3 \| Quite often \| \| 4 \| Very often \| \| 5 \| Always \| |
| 502 | [child_felt_parents_had_time_for_him] | Has your child felt that his/her parent(s) had enough time for him/her? | radio, Required   \| 1 \| Never \| \| --- \| --- \| \| 2 \| Seldom \| \| 3 \| Quite often \| \| 4 \| Very often \| \| 5 \| Always \| |
| 503 | [child_felt_fair_treatment] | Has your child felt that his/her parent(s) treated him/her fairly? | radio, Required   \| 1 \| Never \| \| --- \| --- \| \| 2 \| Seldom \| \| 3 \| Quite often \| \| 4 \| Very often \| \| 5 \| Always \| |
| 504 | [child_talk_what_they_want] | Has your child been able to talk to his/her parent(s) when he/she wanted to? | radio, Required   \| 1 \| Never \| \| --- \| --- \| \| 2 \| Seldom \| \| 3 \| Quite often \| \| 4 \| Very often \| \| 5 \| Always \| |
| 505 | [child_had_enough_money] | Has your child had enough money to do the same things as his/her friends? | radio, Required   \| 1 \| Never \| \| --- \| --- \| \| 2 \| Seldom \| \| 3 \| Quite often \| \| 4 \| Very often \| \| 5 \| Always \| |
| 506 | [child_felt_enough_support_for_expenses] | Has your child felt that he/she had enough money for his/her expenses? | radio, Required   \| 1 \| Never \| \| --- \| --- \| \| 2 \| Seldom \| \| 3 \| Quite often \| \| 4 \| Very often \| \| 5 \| Always \| |
| 507 | [child_enough_money_to_do_things_with_friends] | Does your child feel that he/she has enough money to do things with his/her friends? | radio, Required   \| 1 \| Not at all \| \| --- \| --- \| \| 2 \| Slightly \| \| 3 \| Moderately \| \| 4 \| Very \| \| 5 \| Very much/ Extremely \| |
| 508 | [spent_time_with_friends] | Section Header: *Social support and Peers Friends*  Has your child spent time with his/her friends? | radio, Required   \| 1 \| Never \| \| --- \| --- \| \| 2 \| Seldom \| \| 3 \| Quite often \| \| 4 \| Very often \| \| 5 \| Always \| |
| 509 | [done_things_with_other_children] | Has your child done things with other girls and boys? | radio, Required   \| 1 \| Never \| \| --- \| --- \| \| 2 \| Seldom \| \| 3 \| Quite often \| \| 4 \| Very often \| \| 5 \| Always \| |
| 510 | [child_had_fun_with_friends] | Has your child had fun with his/her friends? | radio, Required   \| 1 \| Never \| \| --- \| --- \| \| 2 \| Seldom \| \| 3 \| Quite often \| \| 4 \| Very often \| \| 5 \| Always \| |
| 511 | [child_help_friends] | Have your child and his/her friends helped each other? | radio, Required   \| 1 \| Never \| \| --- \| --- \| \| 2 \| Seldom \| \| 3 \| Quite often \| \| 4 \| Very often \| \| 5 \| Always \| |
| 512 | [child_talk_friends] | Has your child been able to talk about everything with his/her friends? | radio, Required   \| 1 \| Never \| \| --- \| --- \| \| 2 \| Seldom \| \| 3 \| Quite often \| \| 4 \| Very often \| \| 5 \| Always \| |
| 513 | [child_rely_on_friends] | Has your child been able to rely on his/her friends? | radio, Required   \| 1 \| Never \| \| --- \| --- \| \| 2 \| Seldom \| \| 3 \| Quite often \| \| 4 \| Very often \| \| 5 \| Always \| |
| 514 | [child_happy_at_school] | Section Header: *School School and Learning*  Has your child been happy at school? | radio, Required   \| 1 \| Not at all \| \| --- \| --- \| \| 2 \| Slightly \| \| 3 \| Moderately \| \| 4 \| Very \| \| 5 \| Very much/ Extremely \| |
| 515 | [child_doing_well_school] | Has your child got on well at school? | radio, Required   \| 1 \| Not at all \| \| --- \| --- \| \| 2 \| Slightly \| \| 3 \| Moderately \| \| 4 \| Very \| \| 5 \| Very much/ Extremely \| |
| 516 | [child_satisfied_with_teachers] | Has your child been satisfied with his/her teachers? | radio, Required   \| 1 \| Not at all \| \| --- \| --- \| \| 2 \| Slightly \| \| 3 \| Moderately \| \| 4 \| Very \| \| 5 \| Very much/ Extremely \| |
| 517 | [child_pays_attention] | Has your child been able to pay attention? | radio, Required   \| 1 \| Never \| \| --- \| --- \| \| 2 \| Seldom \| \| 3 \| Quite often \| \| 4 \| Very often \| \| 5 \| Always \| |
| 518 | [child_enjoys_going_to_school] | Has your child enjoyed going to school? | radio, Required   \| 1 \| Never \| \| --- \| --- \| \| 2 \| Seldom \| \| 3 \| Quite often \| \| 4 \| Very often \| \| 5 \| Always \| |
| 519 | [child_get_along_with_teachers] | Has your child got along well with his/her teachers? | radio, Required   \| 1 \| Never \| \| --- \| --- \| \| 2 \| Seldom \| \| 3 \| Quite often \| \| 4 \| Very often \| \| 5 \| Always \| |
| 520 | [child_afraid_other_kids] | Section Header: *Social Acceptance (Bullying) Bullying*  Has your child been afraid of other girls and boys? | radio, Required   \| 1 \| Never \| \| --- \| --- \| \| 2 \| Seldom \| \| 3 \| Quite often \| \| 4 \| Very often \| \| 5 \| Always \| |
| 521 | [children_made_fun_of_child] | Have other girls and boys made fun of your child | radio, Required   \| 1 \| Never \| \| --- \| --- \| \| 2 \| Seldom \| \| 3 \| Quite often \| \| 4 \| Very often \| \| 5 \| Always \| |
| 522 | [child_been_bullied] | Have other girls and boys bullied your child? | radio, Required   \| 1 \| Never \| \| --- \| --- \| \| 2 \| Seldom \| \| 3 \| Quite often \| \| 4 \| Very often \| \| 5 \| Always \| |
