# Supplementary material for: Assessing the impact of tungiasis on children’s quality of life in Kenya
Source: PLoS Negl Trop Dis. 2025 Sep 8;19(9):e0012606. doi: 10.1371/journal.pntd.0012606 (PMC12431661; doi:10.1371/journal.pntd.0012606)

# S1_Fig: Frequency histogram of TLQI scores

**Title:** **Assessing the impact of tungiasis on children’s quality of life in Kenya.**

**Journal:** Quality of Life Research

**Author names:**

Lynne Elson^1,2, *^, Berrick Otieno^1^, Abneel K Matharu^3,4^, Naomi Rithi^3^, Esther Chongwo^5^, Francis Mutebi^6^, Hermann Feldmeier^7^, Jürgen Krücken^4^, Ulrike Fillinger^3,5^, Amina Abubakar^1,5^

**Affiliations:**

^1^ Kenya Medical Research Institute (KEMRI)-Wellcome Trust, Kilifi, Kenya. Orcid ID: 0000-0003-2264-4459.

^2^ Centre for Tropical Medicine and Global Health, Nuffield Department of Medicine, University of Oxford, United Kingdom.

^3^ International Centre of Insect Physiology and Ecology, Mbita, Kenya

^4^ Institute for Parasitology and Tropical Veterinary Medicine, Freie Universität Berlin, Germany

^5^Institute for Human Development, Aga Khan University, Nairobi, Kenya

^6^ School of Veterinary Medicine and Animal Resources, College of Veterinary Medicine, Animal Resources and Biosecurity, Makerere University, Kampala, Uganda

^7^ Institute of Microbiology, Infectious Diseases and Immunology, Charité University Medicine, Berlin, Germany

**Corresponding Author:**

Lynne Elson

Kenya Medical Research Institute (KEMRI)-Wellcome Trust, Hospital Road, Kilifi, Kenya

Email: [lynne.elson@gmail.com](mailto:lynne.elson@gmail.com)

## S1_Fig. Frequency histogram of TLQI scores for all patients.

Maximum possible score 24. Red lines indicate thresholds for quintile grouping.


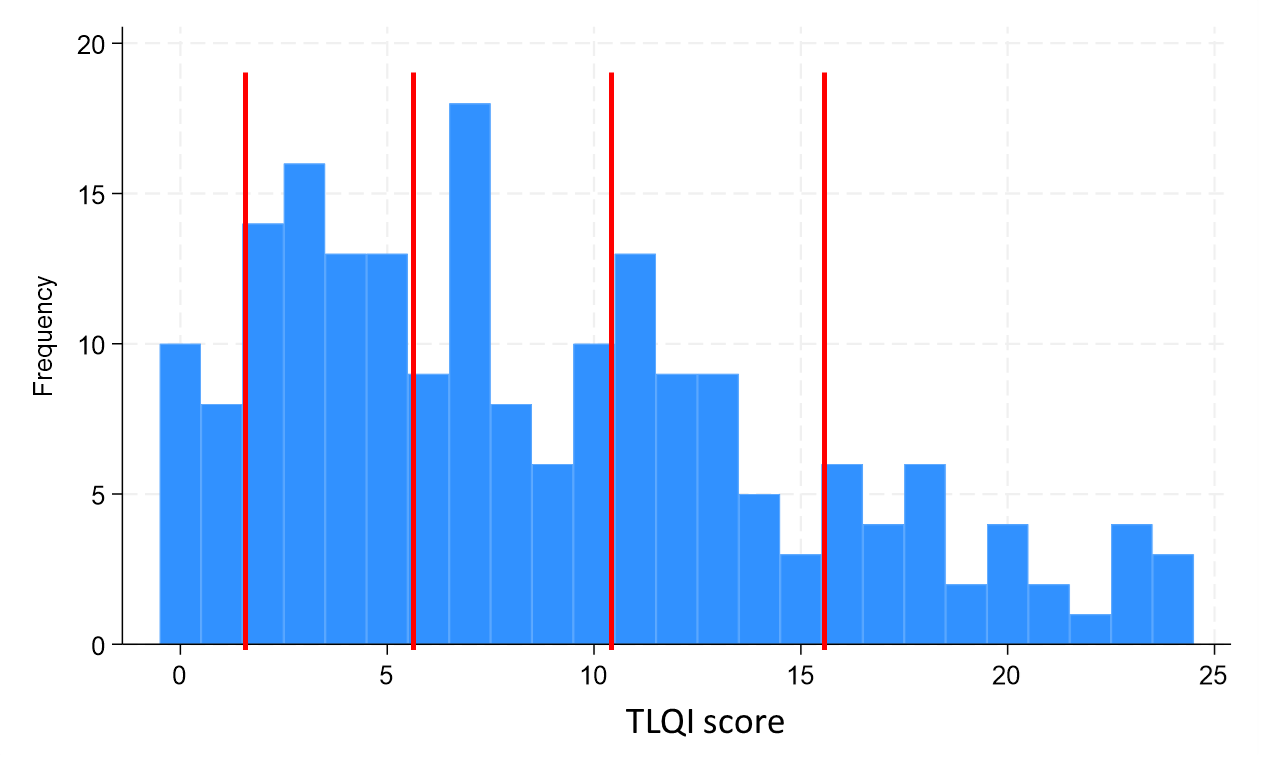

Supplement: S1 Fig — (DOCX) [file pntd.0012606.s003.docx]
