# Supplementary material for: Assessing the impact of tungiasis on children’s quality of life in Kenya
Source: PLoS Negl Trop Dis. 2025 Sep 8;19(9):e0012606. doi: 10.1371/journal.pntd.0012606 (PMC12431661; doi:10.1371/journal.pntd.0012606)
Supplement: S1 Text — (DOCX) [file pntd.0012606.s004.docx]

## S1 Text. Stata code for transforming KIDSREEN52 scores

*<><><><><><><><><><><><><><><><><><><><><><><><><><><><><><><><><><><><><><><><><><>

* Recoding the negatively formulated items (1 to 5 -> high values indicate high HRQoL)

*<><><><><><><><><><><><><><><><><><><><><><><><><><><><><><><><><><><><><><><><><><>

******************physiscal health

recode childs_health (1=5) (2=4) (3=3) (4=2) (5=1),gen (childs_health1)

tab childs_health

tab childs_health1

******************mood & emotions reverse code all the items

recode child_feels_bad_doing_things (1=5) (2=4) (3=3) (4=2) (5=1),gen (child_feels_bad_doing_things1)

tab child_feels_bad_doing_things

tab child_feels_bad_doing_things1

recode child_felt_sad (1=5) (2=4) (3=3) (4=2) (5=1),gen (child_felt_sad1)

tab child_felt_sad

tab child_felt_sad1

recode child_felt_bad_dint_work (1=5) (2=4) (3=3) (4=2) (5=1),gen (child_felt_bad_dint_work1)

tab child_felt_bad_dint_work

tab child_felt_bad_dint_work1

recode child_feels_everything_wrong (1=5) (2=4) (3=3) (4=2) (5=1),gen (child_feels_everything_wrong1)

tab child_feels_everything_wrong

tab child_feels_everything_wrong1

recode child_fed_up (1=5) (2=4) (3=3) (4=2) (5=1),gen (child_fed_up1)

tab child_fed_up

tab child_fed_up1

recode child_felt_lonely (1=5) (2=4) (3=3) (4=2) (5=1),gen (child_felt_lonely1)

tab child_felt_lonely

tab child_felt_lonely1

recode child_felt_under_pressure (1=5) (2=4) (3=3) (4=2) (5=1),gen (child_felt_under_pressure1)

tab child_felt_under_pressure

tab child_felt_under_pressure1

****************** Self perception

recode child_worried_with_looks (1=5) (2=4) (3=3) (4=2) (5=1),gen (child_worried_with_looks1)

tab child_worried_with_looks

tab child_worried_with_looks1

recode child_jealous_of_others_looks (1=5) (2=4) (3=3) (4=2) (5=1),gen (child_jealous_of_others_looks1)

tab child_jealous_of_others_looks

tab child_jealous_of_others_looks1

recode child_wanted_to_change_body (1=5) (2=4) (3=3) (4=2) (5=1),gen (child_wanted_to_change_body1)

tab child_wanted_to_change_body

tab child_wanted_to_change_body1

************ social acceptance (bullying)

recode child_afraid_other_kids (1=5) (2=4) (3=3) (4=2) (5=1),gen (child_afraid_other_kids1)

tab child_afraid_other_kids

tab child_afraid_other_kids1

recode children_made_fun_of_child (1=5) (2=4) (3=3) (4=2) (5=1),gen (children_made_fun_of_child1)

tab children_made_fun_of_child

tab children_made_fun_of_child1

recode child_been_bullied (1=5) (2=4) (3=3) (4=2) (5=1),gen (child_been_bullied1)

tab child_been_bullied

tab child_been_bullied1

*<><><><><><><><><><><><><><><><><><><><><><><><><><><><><><><><><><><><><><><><><><>

* Renaming the variables

*<><><><><><><><><><><><><><><><><><><><><><><><><><><><><><><><><><><><><><><><><><>

* ----------- Physical Well-being -----------

rename childs_health1 KP52PHY1

rename child_fit_well KP52PHY2

rename child_physically_active KP52PHY3

rename child_run_well KP52PHY4

rename child_have_energy KP52PHY5

* ----------- Psychological Well-being -----------

rename child_felt_life_enjoyable KP52PWB1

rename child_felt_pleased_alive KP52PWB2

rename child_satisfied_with_life KP52PWB3

rename child_in_good_moods KP52PWB4

rename child_been_cheerful KP52PWB5

rename child_had_fun KP52PWB6

* ----------- Moods & Emotions -----------

rename child_feels_bad_doing_things1 KP52EMO1

rename child_felt_sad1 KP52EMO2

rename child_felt_bad_dint_work1 KP52EMO3

rename child_feels_everything_wrong1 KP52EMO4

rename child_fed_up1 KP52EMO5

rename child_felt_lonely1 KP52EMO6

rename child_felt_under_pressure1 KP52EMO7

* ----------- Self-Perception -----------

rename child_happy_way_they_are KP52SEL1

rename child_happy_with_clothes KP52SEL2

rename child_worried_with_looks1 KP52SEL3

rename child_jealous_of_others_looks1 KP52SEL4

rename child_wanted_to_change_body1 KP52SEL5

* ----------- Autonomy -----------

rename child_had_enough_time_for_themse KP52AUT1

rename child_do_what_they_want KP52AUT2

rename child_had_enough_time_to_be_outs KP52AUT3

rename child_had_enough_time_meet_frien KP52AUT4

rename child_had_time_choose_what_to_do KP52AUT5

* ----------- Parent Relation & Home Life -----------

rename child_felt_understood_by_parent KP52PAR1

rename child_felt_loved_by_parents KP52PAR2

rename child_happy_at_home KP52PAR3

rename child_felt_parents_had_time_for_ KP52PAR4

rename child_felt_fair_treatment KP52PAR5

rename child_talk_what_they_want KP52PAR6

* ----------- Financial Resources -----------

rename child_had_enough_money KP52FIN1

rename child_felt_enough_support_for_ex KP52FIN2

rename child_enough_money_to_do_things_ KP52FIN3

* ----------- Social Support & Peers -----------

rename spent_time_with_friends KP52SOC1

rename done_things_with_other_children KP52SOC2

rename child_had_fun_with_friends KP52SOC3

rename child_help_friends KP52SOC4

rename child_talk_friends KP52SOC5

rename child_rely_on_friends KP52SOC6

* ----------- School Environment -----------

rename child_happy_at_school KP52SCH1

rename child_doing_well_school KP52SCH2

rename child_satisfied_with_teachers KP52SCH3

rename child_pays_attention KP52SCH4

rename child_enjoys_going_to_school KP52SCH5

rename child_get_along_with_teachers KP52SCH6

* ----------- Social Acceptance (Bullying) -----------

rename child_afraid_other_kids1 KP52BUL1

rename children_made_fun_of_child1 KP52BUL2

rename child_been_bullied1 KP52BUL3

*************************************************************************************

* scoring algorithm for the KIDSCREEN-52 proxy version *

*************************************************************************************

* copyright and intelectual property: The European KIDSCREEN group *

*************************************************************************************

* 1) uses transformed KIDSCREEN item-scores (transformed e.g. by a priori application of the *

* syntax "transform_KIDSCREEN-52_rawdata.SPS") *

* 2) based on the RASCH-Person-Parameter Estimates *

* 3) T-values were computed wich refer to the entire KIDSCREEN survey (escluded were *

* cases older than 18, younger than 8, > 25% missings in KIDSCREEN items, with any *

* missing in the particular scale) *

* 4) for the entire European sample the mean of the T-values is 50, the standard deviation is 10 *

*************************************************************************************

recode KP52PHY1 (5=3) (1/2=4) (3/4=2),gen (KP52PHYc)

tab KP52PHY1

tab KP52PHYc

egen KP52ph_R = rowtotal(KP52PHYc KP52PHY2 KP52PHY3 KP52PHY4 KP52PHY5)

egen KP52pw_R = rowtotal(KP52PWB1 KP52PWB2 KP52PWB3 KP52PWB4 KP52PWB5 KP52PWB6)

egen KP52me_R = rowtotal(KP52EMO1 KP52EMO2 KP52EMO3 KP52EMO4 KP52EMO5 KP52EMO6 KP52EMO7)

egen KP52sp_R = rowtotal(KP52SEL1 KP52SEL2 KP52SEL3 KP52SEL4 KP52SEL5)

egen KP52au_R = rowtotal(KP52AUT1 KP52AUT2 KP52AUT3 KP52AUT4 KP52AUT5)

egen KP52pa_R = rowtotal(KP52PAR1 KP52PAR2 KP52PAR3 KP52PAR4 KP52PAR5 KP52PAR6)

egen KP52fi_R = rowtotal(KP52FIN1 KP52FIN2 KP52FIN3)

egen KP52pe_R = rowtotal(KP52SOC1 KP52SOC2 KP52SOC3 KP52SOC4 KP52SOC5 KP52SOC6)

egen KP52sc_R = rowtotal(KP52SCH1 KP52SCH2 KP52SCH3 KP52SCH4 KP52SCH5 KP52SCH6)

egen KP52bu_R = rowtotal(KP52BUL1 KP52BUL2 KP52BUL3)

egen HRQoL = rowtotal(KP52ph_R KP52pw_R KP52me_R KP52sp_R KP52au_R KP52pa_R KP52fi_R KP52pe_R KP52sc_R KP52bu_R)

sum HRQoL, d

recode KP52ph_R ///

( 5 = -5.365 ) ///

( 6 = -3.806 ) ///

( 7 = -2.984 ) ///

( 8 = -2.439 ) ///

( 9 = -2.01 ) ///

( 10 = -1.641 ) ///

( 11 = -1.302 ) ///

( 12 = -0.976 ) ///

( 13 = -0.642 ) ///

( 14 = -0.283 ) ///

( 15 = 0.114 ) ///

( 16 = 0.559 ) ///

( 17 = 1.049 ) ///

( 18 = 1.574 ) ///

( 19 = 2.116 ) ///

( 20 = 2.671 ) ///

( 21 = 3.273 ) ///

( 22 = 4.015 ) ///

( 23 = 5.318 ), ///

gen (KP52ph_R1)

recode KP52pw_R ///

( 6 = -6.822 ) ///

( 7 = -5.508 ) ///

( 8 = -4.77 ) ///

( 9 = -4.213 ) ///

( 10 = -3.758 ) ///

( 11 = -3.367 ) ///

( 12 = -3.011 ) ///

( 13 = -2.668 ) ///

( 14 = -2.319 ) ///

( 15 = -1.946 ) ///

( 16 = -1.535 ) ///

( 17 = -1.079 ) ///

( 18 = -0.579 ) ///

( 19 = -0.054 ) ///

( 20 = 0.48 ) ///

( 21 = 1.016 ) ///

( 22 = 1.571 ) ///

( 23 = 2.182 ) ///

( 24 = 2.901 ) ///

( 25 = 3.702 ) ///

( 26 = 4.474 ) ///

( 27 = 5.196 ) ///

( 28 = 5.914 ) ///

( 29 = 6.731 ) ///

( 30 = 8.079 ), ///

gen (KP52pw_R1)

recode KP52me_R ///

( 7 = -5.709 ) ///

( 8 = -4.45 ) ///

( 9 = -3.785 ) ///

( 10 = -3.299 ) ///

( 11 = -2.901 ) ///

( 12 = -2.554 ) ///

( 13 = -2.241 ) ///

( 14 = -1.952 ) ///

( 15 = -1.68 ) ///

( 16 = -1.42 ) ///

( 17 = -1.169 ) ///

( 18 = -0.922 ) ///

( 19 = -0.677 ) ///

( 20 = -0.432 ) ///

( 21 = -0.183 ) ///

( 22 = 0.072 ) ///

( 23 = 0.336 ) ///

( 24 = 0.611 ) ///

( 25 = 0.9 ) ///

( 26 = 1.205 ) ///

( 27 = 1.529 ) ///

( 28 = 1.875 ) ///

( 29 = 2.248 ) ///

( 30 = 2.653 ) ///

( 31 = 3.101 ) ///

( 32 = 3.61 ) ///

( 33 = 4.212 ) ///

( 34 = 4.985 ) ///

( 35 = 6.33 ), ///

gen (KP52me_R1)

recode KP52sp_R ///

( 5 = -4.226 ) ///

( 6 = -2.947 ) ///

( 7 = -2.288 ) ///

( 8 = -1.833 ) ///

( 9 = -1.484 ) ///

( 10 = -1.195 ) ///

( 11 = -0.944 ) ///

( 12 = -0.717 ) ///

( 13 = -0.504 ) ///

( 14 = -0.297 ) ///

( 15 = -0.091 ) ///

( 16 = 0.12 ) ///

( 17 = 0.342 ) ///

( 18 = 0.583 ) ///

( 19 = 0.85 ) ///

( 20 = 1.154 ) ///

( 21 = 1.511 ) ///

( 22 = 1.941 ) ///

( 23 = 2.473 ) ///

( 24 = 3.188 ) ///

( 25 = 4.486 ), ///

gen (KP52sp_R1)

recode KP52au_R ///

( 5 = -5.998 ) ///

( 6 = -4.701 ) ///

( 7 = -3.957 ) ///

( 8 = -3.341 ) ///

( 9 = -2.744 ) ///

( 10 = -2.136 ) ///

( 11 = -1.558 ) ///

( 12 = -1.056 ) ///

( 13 = -0.622 ) ///

( 14 = -0.234 ) ///

( 15 = 0.129 ) ///

( 16 = 0.484 ) ///

( 17 = 0.843 ) ///

( 18 = 1.224 ) ///

( 19 = 1.642 ) ///

( 20 = 2.107 ) ///

( 21 = 2.617 ) ///

( 22 = 3.161 ) ///

( 23 = 3.757 ) ///

( 24 = 4.492 ) ///

( 25 = 5.785 ), ///

gen (KP52au_R1)

recode KP52pa_R ///

( 6 = -5.928 ) ///

( 7 = -4.657 ) ///

( 8 = -3.974 ) ///

( 9 = -3.469 ) ///

( 10 = -3.051 ) ///

( 11 = -2.678 ) ///

( 12 = -2.331 ) ///

( 13 = -1.995 ) ///

( 14 = -1.663 ) ///

( 15 = -1.329 ) ///

( 16 = -0.987 ) ///

( 17 = -0.633 ) ///

( 18 = -0.266 ) ///

( 19 = 0.115 ) ///

( 20 = 0.51 ) ///

( 21 = 0.916 ) ///

( 22 = 1.337 ) ///

( 23 = 1.775 ) ///

( 24 = 2.238 ) ///

( 25 = 2.736 ) ///

( 26 = 3.28 ) ///

( 27 = 3.876 ) ///

( 28 = 4.535 ) ///

( 29 = 5.329 ) ///

( 30 = 6.67 ), ///

gen (KP52pa_R1)

recode KP52fi_R ///

( 3 = -5.385 ) ///

( 4 = -3.984 ) ///

( 5 = -3.097 ) ///

( 6 = -2.313 ) ///

( 7 = -1.573 ) ///

( 8 = -0.853 ) ///

( 9 = -0.113 ) ///

( 10 = 0.639 ) ///

( 11 = 1.398 ) ///

( 12 = 2.229 ) ///

( 13 = 3.18 ) ///

( 14 = 4.253 ) ///

( 15 = 5.804 ), ///

gen (KP52fi_R1)

recode KP52pe_R ///

( 6 = -6.422 ) /// ///

( 7 = -5.149 ) /// ///

( 8 = -4.445 ) ///

( 9 = -3.891 ) ///

( 10 = -3.395 ) ///

( 11 = -2.919 ) ///

( 12 = -2.453 ) ///

( 13 = -2.004 ) ///

( 14 = -1.578 ) ///

( 15 = -1.175 ) ///

( 16 = -0.786 ) ///

( 17 = -0.404 ) ///

( 18 = -0.023 ) ///

( 19 = 0.362 ) ///

( 20 = 0.752 ) ///

( 21 = 1.151 ) ///

( 22 = 1.566 ) ///

( 23 = 2.003 ) ///

( 24 = 2.464 ) ///

( 25 = 2.939 ) ///

( 26 = 3.42 ) ///

( 27 = 3.918 ) ///

( 28 = 4.47 ) ///

( 29 = 5.171 ) ///

( 30 = 6.44 ), ///

gen (KP52pe_R1)

recode KP52sc_R ///

( 6 = -6.238 ) ///

( 7 = -4.763 ) ///

( 8 = -3.936 ) ///

( 9 = -3.366 ) ///

( 10 = -2.932 ) ///

( 11 = -2.57 ) ///

( 12 = -2.246 ) ///

( 13 = -1.94 ) ///

( 14 = -1.638 ) ///

( 15 = -1.328 ) ///

( 16 = -0.999 ) ///

( 17 = -0.645 ) ///

( 18 = -0.265 ) ///

( 19 = 0.134 ) ///

( 20 = 0.543 ) ///

( 21 = 0.959 ) ///

( 22 = 1.387 ) ///

( 23 = 1.835 ) ///

( 24 = 2.306 ) ///

( 25 = 2.794 ) ///

( 26 = 3.293 ) ///

( 27 = 3.813 ) ///

( 28 = 4.39 ) ///

( 29 = 5.115 ) ///

( 30 = 6.405 ), ///

gen (KP52sc_R1)

recode KP52bu_R ///

( 3 = -4.785 ) ///

( 4 = -3.441 ) ///

( 5 = -2.65 ) ///

( 6 = -2 ) ///

( 7 = -1.406 ) ///

( 8 = -0.836 ) ///

( 9 = -0.262 ) ///

( 10 = 0.344 ) ///

( 11 = 1.035 ) ///

( 12 = 1.908 ) ///

( 13 = 2.924 ) ///

( 14 = 3.985 ) ///

( 15 = 5.518 ), ///

gen (KP52bu_R1)

gen KP52ph_T = (((KP52ph_R1 - 1.6534) / 1.72649) * 10 + 50)

gen KP52pw_T = (((KP52pw_R1 - 3.1795) / 2.46482) * 10 + 50)

gen KP52me_T = (((KP52me_R1 - 2.8889) / 1.65309) * 10 + 50)

gen KP52sp_T = (((KP52sp_R1 - 1.6327) / 1.36030) * 10 + 50)

gen KP52au_T = (((KP52au_R1 - 2.4396) / 1.86406) * 10 + 50)

gen KP52pa_T = (((KP52pa_R1 - 2.8588) / 1.98338) * 10 + 50)

gen KP52fi_T = (((KP52fi_R1 - 1.7112) / 2.72474) * 10 + 50)

gen KP52pe_T = (((KP52pe_R1 - 1.8590) / 1.98474) * 10 + 50)

gen KP52sc_T = (((KP52sc_R1 - 1.8857) / 2.00823) * 10 + 50)

gen KP52bu_T = (((KP52bu_R1 - 3.8822) / 1.85270) * 10 + 50)

label variable KP52ph_T "proxy 52item Physical international T-values based on RASCH PP"

label variable KP52pw_T "proxy 52item Psychological Wellbeing international T-values based on RASCH PP"

label variable KP52me_T "proxy 52item Moods & Emotions international T-values based on RASCH PP"

label variable KP52sp_T "proxy 52item Self Perception international T-values based on RASCH PP"

label variable KP52au_T "proxy 52item Autonomy international T-values based on RASCH PP"

label variable KP52pa_T "proxy 52item Parents international T-values based on RASCH PP"

label variable KP52fi_T "proxy 52item Financuial international T-values based on RASCH PP"

label variable KP52pe_T "proxy 52item Peers international T-values based on RASCH PP"

label variable KP52sc_T "proxy 52item School international T-values based on RASCH PP"

label variable KP52bu_T "proxy 52item Bullying international T-values based on RASCH PP"

sum KP52ph_T KP52pw_T KP52me_T KP52sp_T KP52au_T KP52pa_T KP52fi_T KP52pe_T KP52sc_T KP52bu_
