# Supplementary material for: Assessing the impact of tungiasis on children’s quality of life in Kenya
Source: PLoS Negl Trop Dis. 2025 Sep 8;19(9):e0012606. doi: 10.1371/journal.pntd.0012606 (PMC12431661; doi:10.1371/journal.pntd.0012606)
Supplement: S3 Table — (DOCX) [file pntd.0012606.s007.docx]

# S3_Table: Participant Distribution by dependent and independent variables and disease status

**Title:** **Assessing the impact of tungiasis on children’s quality of life in Kenya.**

**Journal:** Quality of Life Research

**Author names:**

Lynne Elson^1,2, *^, Berrick Otieno^1^, Abneel K Matharu^3,4^, Naomi Rithi^3^, Esther Chongwo^5^, Francis Mutebi^6^, Hermann Feldmeier^7^, Jürgen Krücken^4^, Ulrike Fillinger^3,5^, Amina Abubakar^1,5^

**Affiliations:**

^1^ Kenya Medical Research Institute (KEMRI)-Wellcome Trust, Kilifi, Kenya. Orcid ID: 0000-0003-2264-4459.

^2^ Centre for Tropical Medicine and Global Health, Nuffield Department of Medicine, University of Oxford, United Kingdom.

^3^ International Centre of Insect Physiology and Ecology, Mbita, Kenya

^4^ Institute for Parasitology and Tropical Veterinary Medicine, Freie Universität Berlin, Germany

^5^Institute for Human Development, Aga Khan University, Nairobi, Kenya

^6^ School of Veterinary Medicine and Animal Resources, College of Veterinary Medicine, Animal Resources and Biosecurity, Makerere University, Kampala, Uganda

^7^ Institute of Microbiology, Infectious Diseases and Immunology, Charité University Medicine, Berlin, Germany

**Corresponding Author:**

Lynne Elson

Kenya Medical Research Institute (KEMRI)-Wellcome Trust, Hospital Road, Kilifi, Kenya

Email: [lynne.elson@gmail.com](mailto:lynne.elson@gmail.com)

## S3_Table: Participant Distribution by dependent and independent variables and disease status

| **DEPENDENT VARIABLES** | | | | | | | |
| --- | --- | --- | --- | --- | --- | --- | --- |
| **Variable** | **source** | **type** | **Missing**  **n** | **Uninfected** | **Infected (mild)** | **Infected (severe)** |  |
| Total population |  | n^1^ | 0 | 199 | 95 | 103 |  |
| Pain (median, IQR^2^) | TLQI^3^ interview | Categorical, Ordinal | 1 |  | 1 (1−2) | 1 (1−3) |  |
| Itching (median, IQR) | TLQI interview | Categorical, Ordinal | 1 |  | 1 (1−3) | 1 (1−3) |  |
| TLQI (median, IQR) | TLQI interview | Count | 1 |  | 7 (3−10.3) | 10 (4−14) |  |
| HR-QoL^4^  (median, IQR) | KIDSCREEN52® interview | Numeric | 0 | 478 (443−545) | 494 (445−587) | 454 (422−522) |  |

^1^ number, ^2^ inter-quartile range, ^3^ tungiasis life quality index, ^4^ general health-related quality of life

| **INDEPENDENT COVARIATES** | | | | | | | | |
| --- | --- | --- | --- | --- | --- | --- | --- | --- |
| **Variable** | **Source** | **Missing**  **n^1^** | **Categories** | **Total**  **n** | **Uninfected**  **n** | **Infected (mild)**  **n** | **Infected (severe)**  **n** |  |
| All |  |  |  | 397 | 199 | 95 | 103 |  |
| County | RF^2^ | 0 | Kwale | 195 | 99 | 42 | 54 |  |
|  |  | 0 | Siaya | 202 | 100 | 53 | 49 |  |
| Age (years) n (mean, sd^3^) | RF | 6 |  | 391  (10.7, 1.9) | 195  (11.0, 2.0) | 95  (10.3, 1.8) | 101  (10.5, 1.7) |  |
| Sex | RF | 2 | Female | 168 | 111 | 34 | 23 |  |
|  |  |  | Male | 227 | 86 | 61 | 80 |  |
| SES^4^  n (mean, sd) | RF | 80 |  | 317  (0.49,0.31) | 158  (0.48, 0.3) | 79  (0.53,0.32) | 80  (0.49,0.33) |  |
| Adults living with | RF | 2 | Both parents | 252 | 131 | 54 | 67 |  |
|  |  |  | Other adults | 143 | 66 | 41 | 36 |  |
| Who cares for child | RF | 3 | Mother | 295 | 150 | 62 | 83 |  |
|  |  |  | Others | 99 | 47 | 33 | 19 |  |
| Who child chooses to go to when unwell | RF | 7 | Parent | 191 | 95 | 46 | 50 |  |
|  |  |  | Other | 199 | 101 | 48 | 50 |  |
| Mother's schooling | RF | 2 | None | 38 | 22 | 5 | 11 |  |
|  |  |  | Don't know | 159 | 72 | 45 | 42 |  |
|  |  |  | Primary | 145 | 77 | 31 | 37 |  |
|  |  |  | Secondary | 53 | 26 | 14 | 13 |  |
| Father's schooling | RF | 4 | None | 13 | 2 | 3 | 8 |  |
|  |  |  | Don't know | 176 | 88 | 41 | 47 |  |
|  |  |  | Primary | 125 | 63 | 31 | 31 |  |
|  |  |  | Secondary | 79 | 43 | 20 | 16 |  |
| Father away a lot | RF | 13 | No | 121 | 60 | 30 | 31 |  |
|  |  |  | Yes | 263 | 132 | 62 | 69 |  |
| Mother away a lot | RF | 6 | No | 274 | 143 | 55 | 76 |  |
|  |  |  | Yes | 117 | 52 | 39 | 26 |  |
| Parents attend school meetings | RF | 3 | Often | 189 | 106 | 38 | 45 |  |
|  |  |  | Not often | 205 | 91 | 57 | 57 |  |
| Family member ill some months | RF | 2 | No | 241 | 122 | 63 | 56 |  |
|  |  |  | Yes | 154 | 75 | 32 | 47 |  |
| Family member has disability | RF | 4 | No | 365 | 180 | 88 | 97 |  |
|  |  |  | Yes | 28 | 16 | 6 | 6 |  |
| HHH sex | HHRF^4^ | 8 | Female | 87 | 44 | 23 | 20 |  |
|  |  |  | Male | 302 | 149 | 71 | 82 |  |
| Caregiver sex | HHRF | 8 | Female | 355 | 183 | 83 | 89 |  |
|  |  |  | Male | 34 | 10 | 11 | 13 |  |
| HHH age n (mean, sd) | HHRF | 8 |  | 389 (48.7,12.8) | 193 (47.8,11.8) | 94  (149.7, 14.0) | 102 (49.5,12.8) |  |
| Age of caregiver n (mean, sd) | HHRF | 8 |  | 389 (39.7,12.1) | 193  (39.7, 12.0) | 94  (41.0, 12.5) | 102  (38.4,11.9) |  |
| Caregiver stress n median (IQR^5^) | Parental stress | 0 |  | 397  (46, 41-50) | 199  (46, 38-49) | 95  (47,41-50) | 103 (48,42-51) |  |
| Caregiver depressed | PHQ9 | 0 | No | 255 | 141 | 58 | 56 |  |
|  |  |  | Yes | 142 | 58 | 37 | 47 |  |
| Child orphaned | HHRF | 0 | No | 371 | 189 | 88 | 94 |  |
|  |  |  | Yes | 26 | 10 | 7 | 9 |  |
| Caregiver spends time with child | HHRF | 8 | A little | 275 | 126 | 73 | 76 |  |
|  |  |  | A lot | 114 | 67 | 21 | 26 |  |
| Child fears family member | RF | 3 | No | 264 | 132 | 64 | 68 |  |
|  |  |  | Yes | 130 | 64 | 31 | 35 |  |
| Caregiver hugs child | HHRF | 8 | No | 279 | 137 | 72 | 70 |  |
|  |  |  | Yes | 110 | 56 | 22 | 32 |  |

^1^ number, ^2^ pupil risk factor interview, ^3^ standard deviation, ^4^ household interview, ^5^ inter-quartile range
