# Supplementary material for: Assessing the impact of tungiasis on children’s quality of life in Kenya
Source: PLoS Negl Trop Dis. 2025 Sep 8;19(9):e0012606. doi: 10.1371/journal.pntd.0012606 (PMC12431661; doi:10.1371/journal.pntd.0012606)
Supplement: S4 Table — (DOCX) [file pntd.0012606.s008.docx]

# S4_Table:

Bivariable mixed effect negative binomial regression analysis for TLQI

**Title:** **Assessing the impact of tungiasis on children’s quality of life in Kenya.**

**Journal:** Quality of Life Research

**Author names:**

Lynne Elson^1,2, *^, Berrick Otieno^1^, Abneel K Matharu^3,4^, Naomi Rithi^3^, Esther Chongwo^5^, Francis Mutebi^6^, Hermann Feldmeier^7^, Jürgen Krücken^4^, Ulrike Fillinger^3,5^, Amina Abubakar^1,5^

**Affiliations:**

^1^ Kenya Medical Research Institute (KEMRI)-Wellcome Trust, Kilifi, Kenya. Orcid ID: 0000-0003-2264-4459.

^2^ Centre for Tropical Medicine and Global Health, Nuffield Department of Medicine, University of Oxford, United Kingdom.

^3^ International Centre of Insect Physiology and Ecology, Mbita, Kenya

^4^ Institute for Parasitology and Tropical Veterinary Medicine, Freie Universität Berlin, Germany

^5^Institute for Human Development, Aga Khan University, Nairobi, Kenya

^6^ School of Veterinary Medicine and Animal Resources, College of Veterinary Medicine, Animal Resources and Biosecurity, Makerere University, Kampala, Uganda

^7^ Institute of Microbiology, Infectious Diseases and Immunology, Charité University Medicine, Berlin, Germany

**Corresponding Author:**

Lynne Elson

Kenya Medical Research Institute (KEMRI)-Wellcome Trust, Hospital Road, Kilifi, Kenya

Email: [lynne.elson@gmail.com](mailto:lynne.elson@gmail.com)

## S4_Table: Bivariable mixed effect negative binomial regression analysis for TLQI

Bivariable, mixed effect negative binomial regression analysis of associations of disease status and potential confounding variables with TLQI using school ID as random effect.

| **Variables** | **Categories** | **N** | **Coefficient** | **95% CI** | | **P** |
| --- | --- | --- | --- | --- | --- | --- |
| Tungiasis severity | Mild | 95 | ref |  | |  |
|  | Severe | 103 | 0.25 | 0.03 | 0.46 | 0.023 |
| Region | Kwale | 96 | ref |  |  |  |
|  | Siaya | 102 | -0.21 | -0.49 | 0.07 | 0.137 |
| Sex | Female | 57 | ref |  |  |  |
|  | Male | 141 | 0.07 | -0.16 | 0.30 | 0.550 |
| Pupil age |  | 196 | 0.03 | -0.03 | 0.09 | 0.303 |
| Adults live with | Both parents | 121 | ref |  |  |  |
|  | Others | 77 | -0.08 | -0.30 | 0.14 | 0.467 |
| Primary caregiver | Mother | 145 | ref |  |  |  |
|  | Other adult | 52 | -0.05 | -0.29 | 0.20 | 0.710 |
| Who child chooses to go to when unwell | Mother | 145 | ref |  |  |  |
|  | Others | 52 | 0.36 | 0.16 | 0.57 | 0.001 |
| Orphaned | No | 371 | ref |  |  |  |
|  | Yes | 26 | 0.16 | -0.23 | 0.54 | 0.431 |
| HHH^6^ sex | Female | 42 | ref |  |  |  |
|  | Male | 151 | 0.09 | -0.17 | 0.35 | 0.484 |
| HHH age |  | 196 | 0.00 | -0.01 | 0.01 | 0.784 |
| Caregiver sex | Female | 169 | ref |  |  |  |
|  | Male | 24 | -0.09 | -0.42 | 0.24 | 0.585 |
| Caregiver age |  | 196 | 0.00 | -0.01 | 0.01 | 0.617 |
| Mother’s education | None | 16 | ref |  |  |  |
|  | Primary | 87 | -0.40 | -0.79 | -0.01 | 0.043 |
|  | Secondary | 68 | -0.31 | -0.70 | 0.07 | 0.110 |
|  | Don’t know | 27 | -0.37 | -0.84 | 0.09 | 0.116 |
| Father’s education | None | 11 | ref |  |  |  |
|  | Primary | 88 | 0.15 | -0.33 | 0.63 | 0.545 |
|  | Secondary | 62 | 0.33 | -0.16 | 0.83 | 0.184 |
|  | Don’t know | 36 | 0.01 | -0.51 | 0.53 | 0.970 |
| Mother away a lot | No | 130 | ref |  |  |  |
|  | Yes | 63 | -0.26 | -0.50 | -0.03 | 0.029 |
| Parents attend school meetings | Often | 83 | ref |  |  |  |
|  | Not often | 114 | -0.31 | -0.52 | -0.09 | 0.005 |
| Parents check homework | Never | 35 | ref |  |  |  |
|  | Sometimes | 163 | -0.01 | -0.29 | 0.27 | 0.969 |
| Family member ill some months | No | 117 | ref |  |  |  |
|  | Yes | 78 | 0.38 | 0.16 | 0.60 | 0.001 |
| Family disability | No | 365 | ref |  |  |  |
|  | Yes | 28 | 0.33 | -0.08 | 0.75 | 0.113 |
| Parental stress score |  | 196 | 0.02 | 0.00 | 0.03 | 0.026 |
| Caregiver depressed | No | 114 | ref |  |  |  |
|  | Yes | 81 | 0.37 | 0.16 | 0.58 | 0.001 |
| Caregiver time spend with child | A little | 146 | ref |  |  |  |
|  | A lot | 47 | -0.13 | -0.39 | 0.13 | 0.319 |
| Child fears a family member | No | 130 | ref |  |  |  |
|  | Yes | 65 | 0.24 | 0.00 | 0.47 | 0.047 |
| Caregiver hugs child | No | 139 | ref |  |  |  |
|  | Yes | 55 | -0.45 | -0.70 | -0.20 | <0.001 |
| Discipline style | Other | 62 | ref |  |  |  |
|  | Beaten | 136 | 0.07 | -0.16 | 0.31 | 0.541 |
