# Supplementary material for: Assessing the impact of tungiasis on children’s quality of life in Kenya
Source: PLoS Negl Trop Dis. 2025 Sep 8;19(9):e0012606. doi: 10.1371/journal.pntd.0012606 (PMC12431661; doi:10.1371/journal.pntd.0012606)
Supplement: S5 Table — (DOCX) [file pntd.0012606.s009.docx]

# S5_Table: linear regression analysis for HR-QoL

**Title:** **Assessing the impact of tungiasis on children’s quality of life in Kenya.**

**Journal:** Quality of Life Research

**Author names:**

Lynne Elson^1,2, *^, Berrick Otieno^1^, Abneel K Matharu^3,4^, Naomi Rithi^3^, Esther Chongwo^5^, Francis Mutebi^6^, Hermann Feldmeier^7^, Jürgen Krücken^4^, Ulrike Fillinger^3,5^, Amina Abubakar^1,5^

**Affiliations:**

^1^ Kenya Medical Research Institute (KEMRI)-Wellcome Trust, Kilifi, Kenya. Orcid ID: 0000-0003-2264-4459.

^2^ Centre for Tropical Medicine and Global Health, Nuffield Department of Medicine, University of Oxford, United Kingdom.

^3^ International Centre of Insect Physiology and Ecology, Mbita, Kenya

^4^ Institute for Parasitology and Tropical Veterinary Medicine, Freie Universität Berlin, Germany

^5^Institute for Human Development, Aga Khan University, Nairobi, Kenya

^6^ School of Veterinary Medicine and Animal Resources, College of Veterinary Medicine, Animal Resources and Biosecurity, Makerere University, Kampala, Uganda

^7^ Institute of Microbiology, Infectious Diseases and Immunology, Charité University Medicine, Berlin, Germany

**Corresponding Author:**

Lynne Elson

Kenya Medical Research Institute (KEMRI)-Wellcome Trust, Hospital Road, Kilifi, Kenya

Email: [lynne.elson@gmail.com](mailto:lynne.elson@gmail.com)

## S5_Table: Bivariable mixed effect linear regression analysis for HR-QoL

Bivariable, mixed effect linear regression analysis of associations of disease status and potential confounding variables with HR-QoL (KIDSCREEN52) using Gaussian distribution, identity link and school ID as random effect.

| **Variables** | **Categories** | **Coefficient** | **95% CI** | | **P** |
| --- | --- | --- | --- | --- | --- |
| Tungiasis status | Uninfected | ref |  |  |  |
|  | Infected | -7.93 | -23.16 | 7.29 | 0.307 |
| Tungiasis severity | Uninfected | ref |  |  |  |
|  | Mild | 6.45 | -12.58 | 25.47 | 0.507 |
|  | Severe | -21.15 | -39.63 | -2.68 | 0.025 |
| Region | Kwale | ref |  |  |  |
|  | Siaya | 33.77 | 14.66 | 52.88 | 0.001 |
| Sex | Girls | ref |  |  |  |
|  | Boys | -9.11 | -24.76 | 6.55 | 0.254 |
| Age |  | -0.65 | -4.81 | 3.50 | 0.758 |
| SES |  | 6.67 | -23.98 | 37.31 | 0.670 |
| Adults child lives with | Both parents | ref |  |  |  |
|  | Others | 3.33 | -12.86 | 19.52 | 0.687 |
| Relationship to caregiver | Parent | ref |  |  |  |
|  | Other | 13.49 | -4.48 | 31.47 | 0.141 |
| Who child chooses to go to when unwell | Mother | ref |  |  |  |
|  | Others | -28.15 | -43.65 | -12.66 | <0.001 |
| Orphaned | No | Ref |  |  |  |
|  | Yes | -14.26 | -46.04 | 17.52 | 0.379 |
| HHH^6^ sex | Female | ref |  |  |  |
|  | Male | 2.63 | -16.57 | 21.83 | 0.788 |
| HHH age |  | 0.00 | -0.61 | 0.62 | 0.992 |
| Caregiver sex | Female | ref |  |  |  |
|  | Male | 12.67 | -15.41 | 40.75 | 0.377 |
| Caregiver age |  | 0.43 | -0.22 | 1.09 | 0.196 |
| Mother education | None | ref |  |  |  |
|  | Don’t know | 12.88 | -15.81 | 41.56 | 0.379 |
|  | Primary | 9.74 | -18.37 | 37.84 | 0.497 |
|  | Secondary | 41.02 | 7.00 | 75.04 | 0.018 |
| Father education | None | ref |  |  |  |
|  | Don’t know | 10.95 | -33.34 | 55.24 | 0.628 |
|  | Primary | 16.47 | -28.31 | 61.26 | 0.471 |
|  | Secondary | 36.02 | -10.22 | 82.25 | 0.127 |
| Father away a lot | No | ref |  |  |  |
|  | Yes | 31.80 | 14.51 | 49.10 | <0.001 |
| Mother away a lot | No | ref |  |  |  |
|  | Yes | 26.28 | 8.86 | 43.69 | 0.003 |
| Family ill months | No | ref |  |  |  |
|  | Yes | -44.94 | -61.03 | -28.85 | <0.001 |
| Family disability | No | ref |  |  |  |
|  | Yes | -21.26 | -51.54 | 9.03 | 0.169 |
| Caregiver stress score |  | -1.19 | -2.17 | -0.21 | 0.017 |
| Caregiver depressed | No | ref |  |  |  |
|  | Yes | -40.21 | -56.46 | -23.97 | <0.001 |
